# Regions of homozygosity as risk factors for multiple myeloma

**DOI:** 10.1111/ahg.12304

**Published:** 2019-02-15

**Authors:** Molly Went, Amit Sud, Ni Li, David C. Johnson, Jonathan S. Mitchell, Martin Kaiser, Richard S. Houlston

**Affiliations:** ^1^ Division of Genetics and Epidemiology The Institute of Cancer Research London UK; ^2^ Division of Molecular Pathology The Institute of Cancer Research London UK

**Keywords:** genetics, multiple myeloma, risk factor

## Abstract

Genomic regions of homozygosity (ROH), detectable in outbred populations, have been implicated as determinants of inherited risk. To examine whether ROH is associated with risk of multiple myeloma (MM), we performed whole‐genome homozygosity analysis using single‐nucleotide polymorphism genotype data from 2,282 MM cases and 5,197 controls, with replication in an additional 878 MM cases and 7,083 controls. Globally, the distribution of ROH between cases and controls was not significantly different. However, one ROH at chromosome 9q21, harboring the B‐cell transcription factor gene *KLF9*, showed evidence of a consistent association and may therefore warrant further investigation as a candidate risk factor for MM. Overall, our analysis provides little support for a homozygosity signature being a significant factor in MM risk.

## 1 INTRODUCTION

Multiple myeloma (MM) is a malignancy of plasma cells that has a significant heritable component as evidenced by the two‐ to fourfold increased risk shown in relatives of MM patients (Altieri, Chen, Bermejo, Castro, & Hemminki, 2006; Palumbo & Anderson, 2011).

Recent genome‐wide association studies (GWAS) have provided the first risk alleles for MM identifying common (minor allele frequency [MAF] >5%) single‐nucleotide polymorphisms (SNPs) at 17 independent loci (Broderick et al., 2011; Chubb et al., 2013; Mitchell et al., 2016; Swaminathan et al., 2015; Went & Sud, 2018). Although these SNPs individually have modest effects on risk (odds ratio [OR] typically <1.5), their identification has yielded novel insights into MM biology, implicating disruption of B‐cell biology and chromatin remodeling pathways in development of the disease (Mitchell et al., 2016).

Whereas much of the heritable risk of MM is likely to be enshrined in such polygenic susceptibility, statistical modeling indicates that a significant fraction of the familial risk will be unexplained (Mitchell et al., 2015). A number of possible explanations for this missing heritability have been proposed including rare and recessive alleles (Manolio et al., 2009; Mitchell et al., 2015). Common regions of homozygosity (ROH), the result of autozygosity (i.e., occurrence of two alleles at the same locus originating from a common ancestor as a consequence of nonrandom mating), have been documented to be detectable at high frequency in outbred populations as a consequence of selective pressure (Ku, Naidoo, Teo, & Pawitan, 2011; Lencz et al., 2007). Searching for ROH on a genome‐wide basis therefore affords a strategy for discovery of recessively acting disease genes and has been exploited in studies of rheumatoid arthritis (Yang, Chang, Liang, Lin, & Wang, 2012), Alzheimer's (Ghani et al., 2015), and early‐onset Parkinson's disease (Simon‐Sanchez et al., 2012). Findings from these studies support the hypothesis that recessive, disease‐predisposing loci not readily detected using a conventional GWAS approach exist (Yang et al., 2012). A possible reason for this apparent failure of conventional GWAS is that the relative risks per locus are too modest and/or that the disease‐associated variants are not in strong linkage disequilibrium (LD) with the tag SNPs.

Although GWAS may therefore have limited ability to identify recessive disease alleles through SNP analyses, such data sets can potentially be exploited in the search for recessively acting disease loci through whole‐genome homozygosity analysis (WGHA). To explore this prospect in MM, we implemented WGHA using SNP genotype data from two GWAS data sets totaling 3,160 cases and 12,280 controls.

## 2 METHODS

### 2.1 Discovery GWAS

For the discovery analysis, we analyzed genotype data from a previously described (Broderick et al., 2011) GWAS, which comprised 2,282 MM cases ascertained through the UK Medical Research Council (MRC) Myeloma‐IX trial (Morgan et al., 2010). Genotype data on 5,197 individuals from the UK Welcome Trust Case‐Control Consortium 2 (WTCCC2) study of individuals from the 1958 British Birth Cohort (58C) (also known as the National Child Development Study) and the UK Blood Service collections served as controls. Cases were genotyped using Illumina Human OmniExpress‐12 (Version 1.0 array; Illumina, San Diego, CA) and controls using Illumina Human 1‐2M‐Duo Custom (Version 1 array chips).

### 2.2 Replication GWAS

For replication, we utilized genotype data generated on an additional series 878 MM cases ascertained through the UK MRC Myeloma‐IX and Myeloma‐XI trials. Controls consisted of (1) 2,976 cancer‐free men recruited by the PRACTICAL Consortium—the UK Genetic Prostate Cancer Study (age <65 years), a study conducted through the Royal Marsden NHS Foundation Trust and SEARCH (Study of Epidemiology & Risk Factors in Cancer Heredity), recruited general practices in East Anglia (2003–2009), and (2) 4,446 cancer‐free women across the United Kingdom, recruited via the Breast Cancer Association Consortium. Both cases and controls were genotyped using Illumina OncoArray.

### 2.3 Ethics

Collection of patient samples and associated medical information was undertaken with ethical review board approval, specifically, for the Myeloma‐IX trial, by the MRC Leukaemia Data Monitoring and Ethics Committee (MREC 02/8/95, ISRCTN68454111), and for the Myeloma‐XI trial, by the Oxfordshire Research Ethics Committee (MREC 17/09/09, ISRCTN49407852). The diagnosis of MM (ICD‐10 C90.0) was established in accordance with World Health Organization guidelines.

### 2.4 Quality control

Both GWAS have previously been subject to stringent quality control (Broderick et al., 2011; Chubb et al., 2013; Mitchell et al., 2016; Swaminathan et al., 2015). Briefly, samples with a low SNP call rate (<95%) were excluded, as were SNPs where <95% of DNA samples generated a genotype at the locus. We excluded individuals of non‐European ancestry (using the HapMap Version 2 CEU, JPT/CHB, and YRI populations as reference). For apparent first‐degree relative pairs, we excluded the control from a case‐control pair; otherwise, we excluded the individual with the lower call rate. SNPs with a MAF <1% or displaying significant deviation from Hardy–Weinberg equilibrium (*p* < 10^−5^) were excluded. Genotypes were imputed to >10 million SNPs using IMPUTE2 (Version 2.3) (Howie, Donnelly, & Marchini, 2009) software in conjunction with a merged reference panel consisting of data from the 1000 Genomes Project (Abecasis et al., 2010) (phase 1 integrated release March 3, 2012) and UK10K, retaining only alleles with an INFO score of >0.8.

### 2.5 Identification of ROH

PLINK (Version 1.9) (Purcell et al., 2007) was used to search for ROH with a specified length, inputted in the homozyg‐SNP parameter, of 73 for the discovery data set and 100 for the replication data set. This resulted in an ROH length that was more than an order of magnitude larger than the mean haploblock size in the human genome without being so large as to be very rare. The likelihood of observing, in the case of the discovery data set, 73 consecutive chance events can be calculated on the basis that mean heterozygosity in the controls was 35%. Given 408,422 SNPs and 7,478 individuals, a minimum length of 57 would be required to produce <5% randomly generated ROH across all subjects (i.e., (1–0.35)^57^ × 408,422 × 7,478 = 0.047). A consequence of LD is that the SNP genotypes are not always independent. Analysis based on PLINK's pairwise LD SNP pruning function revealed 311,773 separable tag groups, representing a 24% reduction of information compared to the original number of SNPs. Thus, ROH of length 73 were used to approximate the degrees of freedom of 57 independent SNP calls.

Following this, an ROH of ≥73 SNPs in length were pruned to only those that occurred in more than 10 individuals. To ensure that a minimum length and number of SNPs in each ROH was maintained, each individual's SNP data were recoded as one if the SNP was in an ROH for that individual and zero otherwise. Each SNP present in fewer than 10 individuals were then recoded to zero before any ROH that were subsequently less than the required number of SNPs in length were removed. This resulted in a list of “common” ROH with a minimum of 73 consecutive ROH calls across 10 or more samples. To investigate association of ROH at a locus, we considered those ROHs with *p*‐value <0.01 in the discovery data set.

### 2.6 Statistics and bioinformatics

We detected ROH using PLINK (Version 1.9), which views a sliding window of SNPs across the genome. We examined broad‐sense ROH, which considers a homozygosity‐rich region containing a small proportion of heterozygotes, the result of genotyping errors, artificial heterozygosity, or mutations. Specifically, our analysis allowed for 2% heterozygotes in each window to prevent an underestimation of the number and size of ROH. The remaining options were set to default values (including allowing five missing calls per window), with the exception of the homozyg‐SNP parameter that was calculated for each data set according to the aforementioned method for defining the ROH length. Statistical analyses were performed using R (Version 3.4) and data was formatted using Perl code. A χ^2^ test was used to compare the distribution of categorical variables between cases and controls. To compare the difference in average number of ROH between cases and controls, we used a Student's *t*‐test. Meta‐analyses were performed in R (Version 3.3.1) using the fixed‐effects inverse‐variance method.

To provide evidence of positive selection at each locus, we obtained iHS, D, and F_st_ metrics from dbPSHP (Li et al., 2014). iHS measures the extent of LD surrounding a locus, with an extreme absolute value being indicative of a positively selected allele (Voight, Kudaravalli, Wen, & Pritchard, 2006). Tajima's D metric infers selection by quantifying the difference between number of segregating sites and average number of nucleotide differences, with a large difference being indicative of selection (Tajima, 1989). F_st_ assesses the proportion of genetic diversity as a result of allele frequency differences within a sample population versus the entire population (Holsinger & Weir, 2009).

## 3 RESULTS

After imposing stringent quality control to GWAS data sets, the discovery GWAS provided genotype data on 2,282 cases and 5,197 controls, and the replication GWAS genotype data on 878 cases and 7,083 controls (Supplementary Tables 1 and 2).

**Figure 1:**
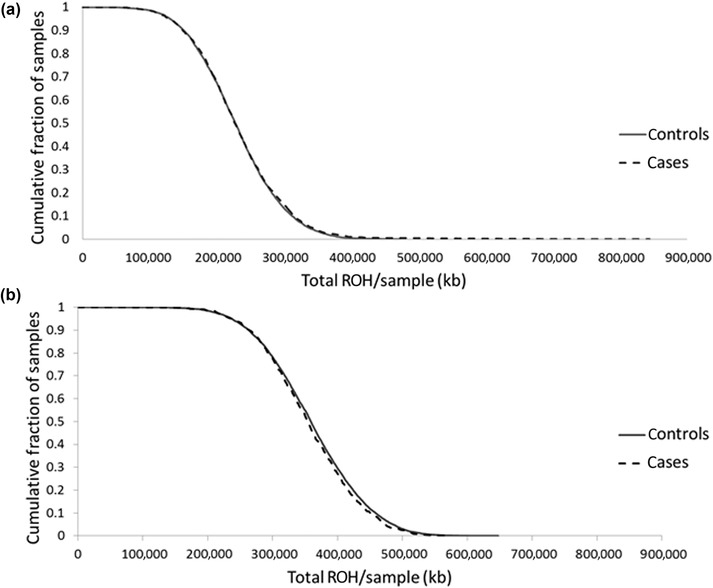
Cumulative distributions of regions of homozygosity (ROH) in cases and controls of (a) discovery, and (b) replication data sets. Each data point represents the cumulative fraction of the samples with the corresponding minimum run of homozygosity

Using PLINK, we found a total of 393 ROH in the discovery data set (Supplementary Table 3, Supplementary Figure 1). The mean number of ROH was lower in cases than in controls (*p* = 0.016), although the mean total length of ROH present was not significantly different (cases 232 Mb, controls 230 Mb; *p* = 0.15; Table 1). The cumulative length of ROH in cases and controls was computed to examine the cumulative distribution in each (Figure 1). ROH encompassing the centromere were seen on chromosomes 1, 2, 3, 4, 5, 6, 7, 8, 10, 11, 12, 16, 17, 18, 19, and 20, including a previously described ROH (Lencz et al., 2007) that bounded the centromere on chromosome 8 and hence served as a control. The longest ROH (44.2 Mb) was the one spanning the centromeric region of chromosome 1. Six regions showed a difference in frequency between cases and controls at a *p* < 0.01 level of significance (Table 2).

**Table 1:** Comparison of regions of homozygosity (ROH) distribution and length in cases versus controls for discovery and replication data sets

	Discovery			Replication		
	Cases	Controls	*p‐*Value	Cases	Controls	*p‐*Value
**Mean number ROH**	15.44	15.7	0.016	21.51	21.69	0.22
**Mean total length kb (SD)**	232 (63)	230 (63)	0.15	355 (72)	360 (75)	0.12

**Table 2:** Results of regions of homozygosity (ROH) associated with multiple myeloma in the discovery data set (*p* < 0.01) and replication data set

	Region (Mb)		ROH ID					No. (%) of samples				*p‐*Value		
Chromosome	Discovery	Replication	Discovery	Replication	Tajima's D	FST	iHS	Discovery cases	Discovery controls	Replication cases	Replication controls	Discovery	Replication	*P*_Meta_
1	88.3–90.5	88.3–107.9	ROH117	ROH18	3.68	0.18	3.03	12 (0.53)	9 (0.17)	137 (16)	1214 (17)	0.0008	0.25	–
5	133.1–134.6	133.0–134.6	ROH219	ROH122	3.92	0.40	2.47	31 (1.14)	38 (0.48)	6 (0.7)	75 (1.1)	0.0014	0.30	–
5	79.2–82.1	79.2–115.1	ROH215	ROH119	3.68	0.28	2.87	26 (1.36)	25 (0.73)	296 (34)	2515 (36)	0.0008	0.29	–
6	149.2–150.5	149.1–150.7	ROH244	ROH145	3.45	0.11	2.79	126 (5.52)	198 (3.81)	4 (0.5)	47 (0.66)	0.0052	0.47	–
9	71.1–74.3	71.4–74.3	ROH296	ROH198	4.04	0.32	2.75	18 (0.79)	13 (0.25)	11 (1.3)	75 (1.1)	0.0080	0.60	0.01
10	73.4–78.0	73.4–79.2	ROH315	ROH223	4.18	0.37	1.81	29 (1.27)	33 (0.63)	231 (26)	1784 (25)	0.0090	0.47	0.18

*Note*. iHS, D, and F_st_ metrics were obtained from dbPSHP (Li et al., 2014). *P*_Meta_ is calculated using an inverse‐variance weighted method.

We sought validation of these findings in the replication GWAS data set. Overall, 362 ROH were found in the replication data set (Supplementary Table 4). As was the case with the discovery GWAS data set, ROH that encompassed the centromere were identified on chromosomes 1, 2, 3, 4, 5, 6, 7, 8, 10, 11, 12, 16, 17, 18, 19, and 20, with the longest ROH (46.4 Mb) spanning the centromere of chromosome 1. The average length of ROH in the replication data set (5.65 Mb), was comparable with that found in the discovery (5.07 Mb) series. In contrast to the discovery series, the average number of ROH in the replication data set was not significantly different between cases and controls (Table 1). Furthermore, the mean total length of ROH was not significantly different (cases: 355 Mb, controls: 360 Mb; *p* = 0.12; Table 1). The cumulative distribution provided a demonstration of the comparability in distribution of ROH between cases and controls (Figure 1).

Of the six candidate ROH linked to MM risk in the discovery GWAS, two ROH located at 9q21 and 10q22 showed a consistent direction of effect, with 9q21 having a comparable frequency (Table 2). The combined *p‐*values for the consensus ROH regions at 9q21 and 10q22 (Figure 2), were 0.01 and 0.18, respectively (Table 2). Although the 9q21 association is not significant after adjusting for multiple testing, such correction can result in a type II error. Support for positive selection of this region is provided by the associated high D and iHS metrics (Table 2). Intriguingly, the consensus region for the ROH at 9q21, contains the gene encoding the transcription factor KLF9. Decreased levels of KLF9 have been implicated in a hyperproliferative B‐cell response and defective Ig production (Good & Tangye, 2007; Savignac et al., 2010) and a predictive marker of sensitivity to bortezomib treatment in MM (Mannava et al., 2012; Ri, 2016).

Figure 2Plot of overlapping region of discovery (navy) and replication regions of homozygosity (light blue) on (a) chromosome 9, and (b) chromosome 10. Coordinates are from the National Center for Biotechnology Information Build 37 human genome [Color figure can be viewed at wileyonlinelibrary.com]

**Figure:**
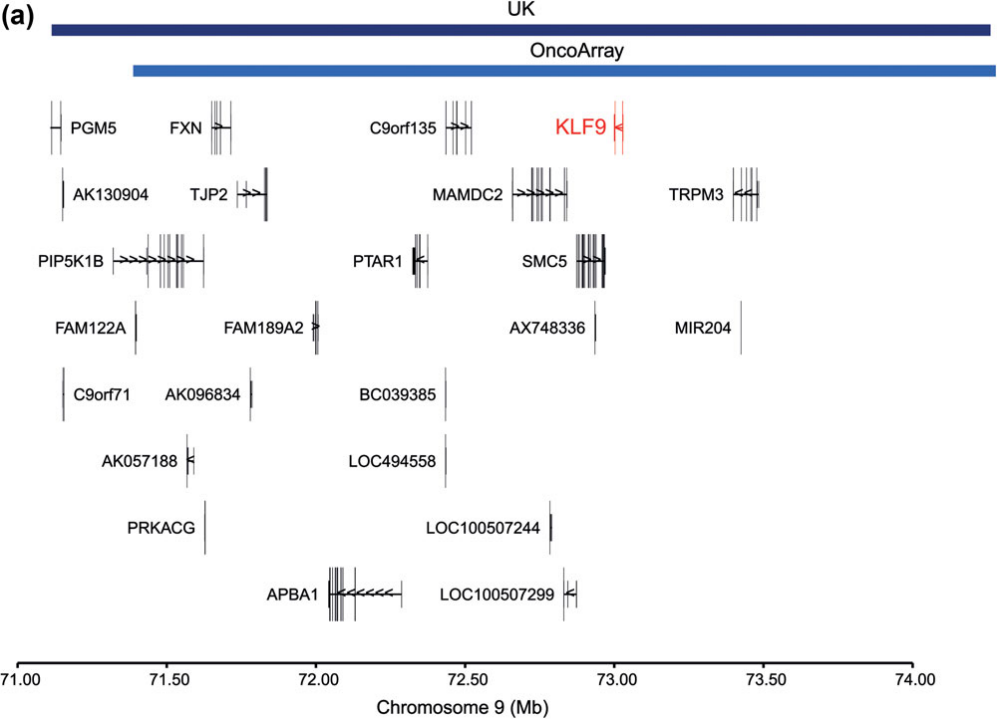

**Figure:**
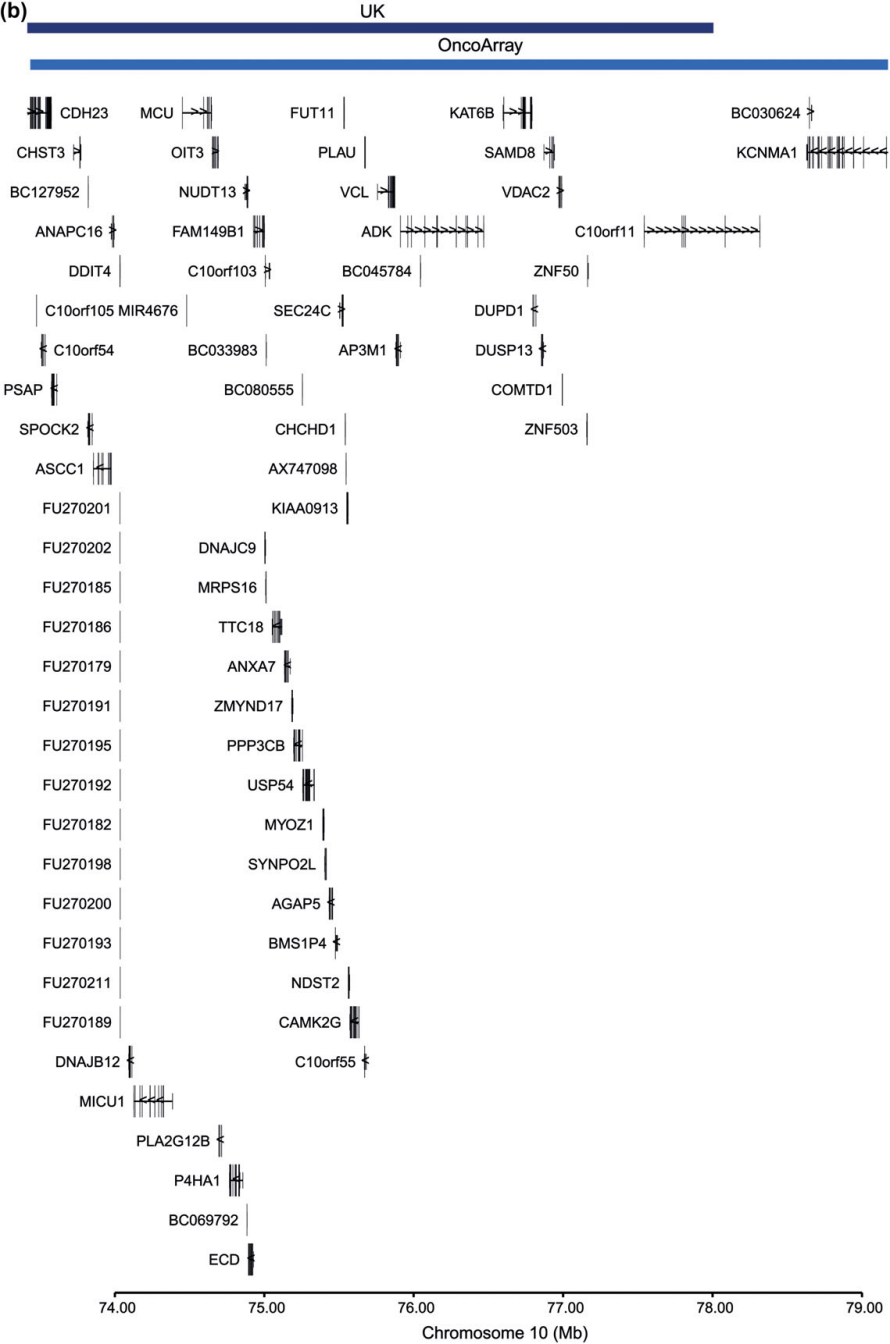

## 4 DISCUSSION

Recent studies have suggested that analysis of genomic ROH may provide evidence of cancer risk (Assie, Laframboise, Platzer, & Eng, 2008; Thomsen et al., 2016a, 2016b; Wang et al., 2013). Specifically, that interrogation of locus‐specific ROH can provide a strategy to identify recessive disease‐causing variants. Generally, the strategy has been applied to populations characterized by an increased degree of inbreeding (Feldman, Lee, & Seligman, 1976), although there is evidence that the success in such populations may be attributed to confounding factors (Abdellaoui et al., 2013, 2015; Ceballos, Joshi, Clark, Ramsay, & Wilson, 2018; Mitchell et al., 2016). More recently, the strategy has been applied in outbred populations; however, few of the reported analyses have sought replication of study findings. It is notable that in a recent WGHA of schizophrenia risk, attempts to validate previous findings have failed despite a well‐powered replication analysis (Mitchell et al., 2016).

Here, we have sought to mitigate against confounding factors or the winner's curse (Palmer & Pe'er, 2017) by investigating the homozygosity signature of SNP genotype data first in a discovery and then in an independent replication data set, totaling 3,160 MM cases and 12,280 population‐matched controls. A further strength of our analysis is the stringent quality control applied to each of the data sets; notably, both had no demonstrable evidence of population stratification. Finally, to search for meaningful ROH and avoid inflation because of correlated SNPs, we adjusted computed ROH length to account for the fraction of SNPs that were in tagged regions.

Our discovery WGHA and replication provided no evidence of an association between global or individual ROH and MM risk. While not statistically significant after correction for multiple testing, an ROH at 9q21 showed a consistent direction of effect. The consensus region at 9p21 implicates the transcription factor KLF9, which, when given a plausible biological role in MM pathogenesis, may warrant further investigation.

In conclusion, our analysis provides little support for a homozygosity signature being a significant factor in MM risk. However, we cannot exclude the possibility of recessive alleles influencing MM risk.

## CONFLICTS OF INTEREST

The authors declare no conflict of interest.

## Supporting information

Table S1 Details of the quality control filters applied to each data set
Table S2 Details of the quality control filters applied to each data set
Table S3 Details of the ROH found in the discovery data set
Table S4 Details of the ROH found in the replication data set
Figure S1 Location within the autosome and frequency of the ROH found in (a) discovery, and (b) replication data set.

## References

[ahg12304-bib-0001] Abdellaoui, A. , Hottenga, J. J. , Xiao, X. , Scheet, P. , Ehli, E. A. , Davies, G. E. , … Boomsma, D. I. (2013). Association between autozygosity and major depression: Stratification due to religious assortment. Behavior Genetics, 43, 455–467. 23978897 10.1007/s10519-013-9610-1 PMC3827717

[ahg12304-bib-0002] Abdellaoui, A. , Hottenga, J. J. , Willemsen, G. , Bartels, M. , Van Beijsterveldt, T. , Ehli, E. A. , … Boomsma, D. I. (2015). Educational attainment influences levels of homozygosity through migration and assortative mating. PLoS One, 10, e0118935. 25734509 10.1371/journal.pone.0118935 PMC4347978

[ahg12304-bib-0003] Abecasis, G. R. , Altshuler, D. , Auton, A. , Brooks, L. D. , Durbin, R. M. , Gibbs, R. A. , … Mcvean, G. A. (2010). A map of human genome variation from population‐scale sequencing. Nature, 467, 1061–1073. 20981092 10.1038/nature09534 PMC3042601

[ahg12304-bib-0004] Altieri, A. , Chen, B. , Bermejo, J. L. , Castro, F. , & Hemminki, K. (2006). Familial risks and temporal incidence trends of multiple myeloma. European Journal of Cancer, 42, 1661–1670. 16753294 10.1016/j.ejca.2005.11.033

[ahg12304-bib-0005] Assie, G. , Laframboise, T. , Platzer, P. , & Eng, C. (2008). Frequency of germline genomic homozygosity associated with cancer cases. JAMA, 299, 1437–1445. 18364486 10.1001/jama.299.12.1437

[ahg12304-bib-0006] Broderick, P. , Chubb, D. , Johnson, D. C. , Weinhold, N. , Forsti, A. , Lloyd, A. , … Houlston, R. S. (2011). Common variation at 3p22.1 and 7p15.3 influences multiple myeloma risk. Nature Genetics, 44, 58–61. 22120009 10.1038/ng.993 PMC5108406

[ahg12304-bib-0007] Ceballos, F. C. , Joshi, P. K. , Clark, D. W. , Ramsay, M. , & Wilson, J. F. (2018). Runs of homozygosity: Windows into population history and trait architecture. Nature Reviews. Genetics, 19, 220–234. 10.1038/nrg.2017.109 29335644

[ahg12304-bib-0008] Chubb, D. , Weinhold, N. , Broderick, P. , Chen, B. , Johnson, D. C. , Forsti, A. , … Goldschmidt, H. (2013). Common variation at 3q26.2, 6p21.33, 17p11.2 and 22q13.1 influences multiple myeloma risk. Nature Genetics, 45, 1221–1225. 23955597 10.1038/ng.2733 PMC5053356

[ahg12304-bib-0009] Feldman, J. G. , Lee, S. L. , & Seligman, B. (1976). Occurrence of acute leukemia in females in a genetically isolated population. Cancer, 38, 2548–2550. 1069604 10.1002/1097-0142(197612)38:6<2548::aid-cncr2820380644>3.0.co;2-y

[ahg12304-bib-0010] Ghani, M. , Reitz, C. , Cheng, R. , Vardarajan, B. N. , Jun, G. , Sato, C. , … Rogaeva, E. (2015). Association of long runs of homozygosity with Alzheimer disease among African American individuals. JAMA Neurology, 72, 1313–1323. 26366463 10.1001/jamaneurol.2015.1700 PMC4641052

[ahg12304-bib-0011] Good, K. L. , & Tangye, S. G. (2007). Decreased expression of Kruppel‐like factors in memory B cells induces the rapid response typical of secondary antibody responses. Proceedings of the National Academy of Sciences, USA, 104, 13420–13425. 10.1073/pnas.0703872104 PMC1948953 17673551

[ahg12304-bib-0012] Holsinger, K. E. , & Weir, B. S. (2009). Genetics in geographically structured populations: Defining, estimating and interpreting *F* _ST_ . Nature Reviews. Genetics, 10, 639–650. 10.1038/nrg2611 PMC4687486 19687804

[ahg12304-bib-0013] Howie, B. N. , Donnelly, P. , & Marchini, J. (2009). A flexible and accurate genotype imputation method for the next generation of genome‐wide association studies. PLoS Genetics, 5, e1000529. 19543373 10.1371/journal.pgen.1000529 PMC2689936

[ahg12304-bib-0014] Ku, C. S. , Naidoo, N. , Teo, S. M. , & Pawitan, Y. (2011). Regions of homozygosity and their impact on complex diseases and traits. Human Genetics, 129, 1–15. 21104274 10.1007/s00439-010-0920-6

[ahg12304-bib-0015] Lencz, T. , Lambert, C. , Derosse, P. , Burdick, K. E. , Morgan, T. V. , Kane, J. M. , … Malhotra, A. K. (2007). Runs of homozygosity reveal highly penetrant recessive loci in schizophrenia. Proceedings of the National Academy of Sciences, USA, 104, 19942–19947. 10.1073/pnas.0710021104 PMC2148402 18077426

[ahg12304-bib-0016] Li, M. J. , Wang, L. Y. , Xia, Z. , Wong, M. P. , Sham, P. C. , & Wang, J. (2014). dbPSHP: A database of recent positive selection across human populations. Nucleic Acids Research, 42, D910–D916. 24194603 10.1093/nar/gkt1052 PMC3965004

[ahg12304-bib-0017] Mannava, S. , Zhuang, D. , Nair, J. R. , Bansal, R. , Wawrzyniak, J. A. , Zucker, S. N. , … Nikiforov, M. A. (2012). KLF9 is a novel transcriptional regulator of bortezomib‐ and LBH589‐induced apoptosis in multiple myeloma cells. Blood, 119, 1450–1458. 22144178 10.1182/blood-2011-04-346676 PMC3286209

[ahg12304-bib-0018] Manolio, T. A. , Collins, F. S. , Cox, N. J. , Goldstein, D. B. , Hindorff, L. A. , Hunter, D. J. , … Visscher, P. M. (2009). Finding the missing heritability of complex diseases. Nature, 461, 747–753. 19812666 10.1038/nature08494 PMC2831613

[ahg12304-bib-0019] Mitchell, J. S. , Johnson, D. C. , Litchfield, K. , Broderick, P. , Weinhold, N. , Davies, F. E. , … Houlston, R. S. (2015). Implementation of genome‐wide complex trait analysis to quantify the heritability in multiple myeloma. Scientific Reports, 5, 12473. 26208354 10.1038/srep12473 PMC4513545

[ahg12304-bib-0020] Mitchell, J. S. , Li, N. , Weinhold, N. , Forsti, A. , Ali, M. , Van Duin, M. , … Houlston, R. S. (2016). Genome‐wide association study identifies multiple susceptibility loci for multiple myeloma. Nature Communications, 7, 12050. 10.1038/ncomms12050 PMC4932178 27363682

[ahg12304-bib-0021] Morgan, G. J. , Davies, F. E. , Gregory, W. M. , Cocks, K. , Bell, S. E. , Szubert, A. J. , … Child, J. A. (2010). First‐line treatment with zoledronic acid as compared with clodronic acid in multiple myeloma (MRC Myeloma IX): A randomised controlled trial. Lancet, 376, 1989–1999. 21131037 10.1016/S0140-6736(10)62051-X PMC3639680

[ahg12304-bib-0022] Palmer, C. , & Pe'er, I. (2017). Statistical correction of the Winner's Curse explains replication variability in quantitative trait genome‐wide association studies. PLoS Genetics, 13, e1006916. 28715421 10.1371/journal.pgen.1006916 PMC5536394

[ahg12304-bib-0023] Palumbo, A. , & Anderson, K. (2011). Multiple myeloma. New England Journal of Medicine, 364, 1046–1060. 21410373 10.1056/NEJMra1011442

[ahg12304-bib-0024] Purcell, S. , Neale, B. , Todd‐Brown, K. , Thomas, L. , Ferreira, M. A. , Bender, D. , … Sham, P. C. (2007). PLINK: A tool set for whole‐genome association and population‐based linkage analyses. American Journal of Human Genetics, 81, 559–575. 17701901 10.1086/519795 PMC1950838

[ahg12304-bib-0025] Ri, M. (2016). Endoplasmic‐reticulum stress pathway‐associated mechanisms of action of proteasome inhibitors in multiple myeloma. International Journal of Hematology, 104, 273–280. 27169614 10.1007/s12185-016-2016-0

[ahg12304-bib-0026] Savignac, M. , Mellstrom, B. , Bebin, A. G. , Oliveros, J. C. , Delpy, L. , Pinaud, E. , & Naranjo, J. R. (2010). Increased B cell proliferation and reduced Ig production in DREAM transgenic mice. Journal of Immunology, 185, 7527–7536. 10.4049/jimmunol.1000152 21059893

[ahg12304-bib-0027] Simon‐Sanchez, J. , Kilarski, L. L. , Nalls, M. A. , Martinez, M. , Schulte, C. , Holmans, P. , … Morris, H. R. (2012). Cooperative genome‐wide analysis shows increased homozygosity in early onset Parkinson's disease. PLoS One, 7, e28787. 22427796 10.1371/journal.pone.0028787 PMC3299635

[ahg12304-bib-0028] Swaminathan, B. , Thorleifsson, G. , Joud, M. , Ali, M. , Johnsson, E. , Ajore, R. , … Nilsson, B. (2015). Variants in ELL2 influencing immunoglobulin levels associate with multiple myeloma. Nature Communications, 6, 7213. 10.1038/ncomms8213 PMC4455110 26007630

[ahg12304-bib-0029] Tajima, F. (1989). Statistical method for testing the neutral mutation hypothesis by DNA polymorphism. Genetics, 123, 585–595. 2513255 10.1093/genetics/123.3.585 PMC1203831

[ahg12304-bib-0030] Thomsen, H. , Chen, B. , Figlioli, G. , Elisei, R. , Romei, C. , Cipollini, M. , … Forsti, A. (2016a). Runs of homozygosity and inbreeding in thyroid cancer. BMC Cancer, 16, 227. 26984635 10.1186/s12885-016-2264-7 PMC4794977

[ahg12304-bib-0031] Thomsen, H. , Inacio Da Silva Filho, M. , Fuchs, M. , Ponader, S. , Pogge Von Strandmann, E. , Eisele, L. , … Forsti, A. (2016b). Evidence of inbreeding in Hodgkin lymphoma. PLoS One, 11, e0154259. 27123581 10.1371/journal.pone.0154259 PMC4849743

[ahg12304-bib-0032] Voight, B. F. , Kudaravalli, S. , Wen, X. , & Pritchard, J. K. (2006). A map of recent positive selection in the human genome. PLoS Biology, 4, e72. 16494531 10.1371/journal.pbio.0040072 PMC1382018

[ahg12304-bib-0033] Wang, C. , Xu, Z. , Jin, G. , Hu, Z. , Dai, J. , Ma, H. , … Shen, H. (2013). Genome‐wide analysis of runs of homozygosity identifies new susceptibility regions of lung cancer in Han Chinese. Journal of Biomedical Research, 27, 208–214. 23720676 10.7555/JBR.27.20130017 PMC3664727

[ahg12304-bib-0034] Went, M. , & Sud, A. (2018). Identification of multiple risk loci and regulatory mechanisms influencing susceptibility to multiple myeloma. Nature Communications, 9, 3707. 10.1038/s41467-018-04989-w PMC6137048 30213928

[ahg12304-bib-0035] Yang, H. C. , Chang, L. C. , Liang, Y. J. , Lin, C. H. , & Wang, P. L. (2012). A genome‐wide homozygosity association study identifies runs of homozygosity associated with rheumatoid arthritis in the human major histocompatibility complex. PLoS One, 7, e34840. 22536334 10.1371/journal.pone.0034840 PMC3335047

