# Supplementary material for: Regions of homozygosity as risk factors for multiple myeloma
Source: Ann Hum Genet. 2019 Feb 15;83(4):231–8. doi: 10.1111/ahg.12304 (PMC6563058; doi:10.1111/ahg.12304)
Supplement: Supplementary file 1 — Table S1 Details of the quality control filters applied to each data set Table S2 Details of the quality control filters applied to each data set Table S3 Details of the ROH found in the discovery data set Table S4 Details of the ROH found in the replication data set Figure S1 Location within the autosome and frequency of the ROH found in (a) discovery, and (b) replication data set. [file AHG-83-231-s001.docx]

**Regions of homozygosity as risk factors for multiple myeloma**

**Supplementary Tables and Figures**

Went M^1,2^, Sud A^1^, Ni Li,  Johnson DC^2^, Mitchell JS^1^, Kaiser M^2^, Houlston RS^1^

1. Division of Genetics and Epidemiology, The Institute of Cancer Research, London, UK.
2. Division of Molecular Pathology, The Institute of Cancer Research, London, UK.

|  | **Discovery** | | **Replication** | |
| --- | --- | --- | --- | --- |
|  | **Cases** | **Controls** | **Cases** | **Controls** |
|  |  |  |  |  |
| Pre-QC | 2,329 | 5,199 | 931 | 7,519 |
|  |  |  |  |  |
| Sex discrepancy | 10 | 0 | 6 | 8 |
| Call rate fail | 1 | 0 | 1 | 1 |
| Heterozygosity rate | NA | NA | 5 | 7 |
| Related Individuals | 2 | 2 | 3 | 68 |
| Non-European Ancestry | 34 | 0 | 44 | 364 |
|  |  |  |  |  |
| Post-QC | 2,282 | 5,197 | 878 | 7,083 |

**Supplementary Table 1: Details of the quality control filters applied to each dataset.** For the replication dataset, those with low call rate (<95%), of non-European descent, related individuals or those with a sex discrepancy were excluded. The discovery dataset has been previously reported comprehensive details on QC (Broderick et al., 2011).

|  | **Discovery** | **Replication** |
| --- | --- | --- |
|  |  |  |
|  |  |  |
| Pre-QC | 409,429 | 459,068 |
|  |  |  |
| Call rate fail | 997 | 6,851 |
| HWE fail | 7 | 12 |
| MAF < 0.01 | 3 | 73,239 |
|  |  |  |
| Post-QC | 408,422 | 378,966 |
|  |  |  |
| Imputed (filtered) | 8,517,071 | 3,874,958 |

**Supplementary Table 2: Details of the quality control filters applied to each dataset.** For the replication, genotyped SNPs with a low call rate (<95%), minor allele frequency (MAF) <1% or displaying significant deviation from Hardy-Weinberg equilibrium (i.e. *P* < 10^-5^ ) were excluded. Imputed SNPs with information score <0.8 and MAF <1% were excluded. The discovery dataset has been previously reported comprehensive details on QC (Broderick et al., 2011).

| ROH ID | No. of Cases | No. of Controls | χ² | *P-*value | Chromosome | Start bp (NCBI37) | End bp (NCBI37) | Length | Annotation |
| --- | --- | --- | --- | --- | --- | --- | --- | --- | --- |
| ROH1 | 15 | 30 | 0.169953473 | 0.6801532 | 1 | 798959 | 2211849 | 1412890 |  |
| ROH2 | 13 | 24 | 0.374825032 | 0.5403859 | 4 | 141879419 | 153629791 | 11750372 |  |
| ROH3 | 12 | 14 | 3.010751464 | 0.08271394 | 9 | 87802989 | 89240136 | 1437147 |  |
| ROH4 | 58 | 99 | 3.127747846 | 0.07697 | 16 | 3023605 | 3790186 | 766581 |  |
| ROH5 | 11 | 10 | 4.75019155 | 0.02929503 | 17 | 15099941 | 21707060 | 6607119 |  |
| ROH6 | 37 | 58 | 3.229177498 | 0.07233701 | 18 | 12040933 | 13271319 | 1230386 |  |
| ROH7 | 9 | 20 | 0.003746974 | 0.9511899 | 18 | 68107719 | 69234447 | 1126728 |  |
| ROH8 | 11 | 17 | 1.020381866 | 0.3124284 | 20 | 11728425 | 12250349 | 521924 |  |
| ROH9 | 89 | 176 | 1.223494474 | 0.2686759 | 21 | 23264430 | 24929472 | 1665042 |  |
| ROH10 | 43 | 88 | 0.336250444 | 0.5620016 | 1 | 5904718 | 7486317 | 1581599 |  |
| ROH11 | 32 | 70 | 0.036110406 | 0.8492877 | 1 | 39219090 | 41319146 | 2100056 |  |
| ROH12 | 6 | 8 | 1.008197536 | 0.315335 | 1 | 157997676 | 158656973 | 659297 |  |
| ROH13 | 10 | 32 | 0.894947052 | 0.3441402 | 1 | 242695639 | 244268142 | 1572503 |  |
| ROH14 | 281 | 570 | 2.84853398 | 0.0914573 | 2 | 47538477 | 64719236 | 17180759 |  |
| ROH15 | 146 | 344 | 0.126849951 | 0.7217207 | 2 | 133883098 | 169658783 | 35775685 |  |
| ROH16 | 14 | 36 | 0.149822116 | 0.6987054 | 2 | 228696351 | 229883422 | 1187071 |  |
| ROH17 | 1 | 17 | 5.300375403 | 0.02132083 | 3 | 25460596 | 29018088 | 3557492 |  |
| ROH18 | 13 | 15 | 3.358143642 | 0.06687341 | 3 | 133604743 | 139696209 | 6091466 |  |
| ROH19 | 19 | 58 | 1.250114871 | 0.2635305 | 4 | 8562668 | 11267586 | 2704918 |  |
| ROH20 | 290 | 634 | 0.379114077 | 0.5380777 | 4 | 156677592 | 169425339 | 12747747 |  |
| ROH21 | 21 | 44 | 0.099709887 | 0.7521781 | 5 | 53925541 | 55660509 | 1734968 |  |
| ROH22 | 352 | 785 | 0.12611338 | 0.7224964 | 5 | 134893439 | 141391532 | 6498093 |  |
| ROH23 | 449 | 924 | 3.80424796 | 0.05112273 | 6 | 15032722 | 16235577 | 1202855 |  |
| ROH24 | 8 | 6 | 4.691670209 | 0.03030917 | 6 | 107216553 | 124156824 | 16940271 |  |
| ROH25 | 10 | 13 | 1.829392751 | 0.1761993 | 7 | 139113 | 2600076 | 2460963 |  |
| ROH26 | 9 | 11 | 1.985283247 | 0.1588349 | 7 | 37908779 | 41524795 | 3616016 |  |
| ROH27 | 9 | 12 | 1.51371573 | 0.218573 | 7 | 156381107 | 156952133 | 571026 |  |
| ROH28 | 508 | 1215 | 1.117261385 | 0.2905089 | 8 | 73999481 | 96366766 | 22367285 |  |
| ROH29 | 57 | 115 | 0.573212067 | 0.4489853 | 9 | 16540987 | 17488111 | 947124 |  |
| ROH30 | 26 | 54 | 0.150719009 | 0.6978492 | 9 | 90799534 | 92167606 | 1368072 |  |
| ROH31 | 21 | 48 | 0.00019636 | 0.9888197 | 10 | 17081153 | 24653585 | 7572432 |  |
| ROH32 | 223 | 465 | 1.291039704 | 0.2558564 | 10 | 95395727 | 99603001 | 4207274 |  |
| ROH33 | 5 | 10 | 0.05642346 | 0.8122408 | 11 | 36898350 | 43956936 | 7058586 |  |
| ROH34 | 6 | 18 | 0.345032575 | 0.5569386 | 12 | 8770580 | 10094841 | 1324261 |  |
| ROH35 | 12 | 18 | 1.278867852 | 0.2581096 | 12 | 76421254 | 92388461 | 15967207 |  |
| ROH36 | 10 | 10 | 3.592044363 | 0.05805678 | 13 | 37440146 | 40498828 | 3058682 |  |
| ROH37 | 30 | 47 | 2.619438317 | 0.1055618 | 14 | 27262305 | 30292318 | 3030013 |  |
| ROH38 | 4 | 9 | 0.000406091 | 0.9839224 | 14 | 101781338 | 104673221 | 2891883 |  |
| ROH39 | 25 | 52 | 0.140310214 | 0.7079728 | 15 | 70431773 | 79250141 | 8818368 |  |
| ROH40 | 31 | 43 | 4.56496548 | 0.03263233 | 16 | 13894417 | 17576804 | 3682387 |  |
| ROH41 | 24 | 45 | 0.599035051 | 0.4389464 | 16 | 17650546 | 22859621 | 5209075 |  |
| ROH42 | 230 | 539 | 0.147056707 | 0.7013641 | 16 | 27918169 | 31957368 | 4039199 |  |
| ROH43 | 205 | 475 | 0.04701226 | 0.8283462 | 16 | 35102323 | 49167500 | 14065177 |  |
| ROH44 | 20 | 31 | 1.83466572 | 0.1755774 | 16 | 55789570 | 56917953 | 1128383 |  |
| ROH45 | 68 | 151 | 0.030813465 | 0.8606572 | 16 | 58743454 | 65928153 | 7184699 |  |
| ROH46 | 322 | 779 | 0.975900359 | 0.323213 | 16 | 65985899 | 73263508 | 7277609 |  |
| ROH47 | 7 | 10 | 0.913958196 | 0.3390664 | 16 | 74073632 | 77737858 | 3664226 |  |
| ROH48 | 5 | 8 | 0.388141833 | 0.5332778 | 16 | 88633347 | 90141355 | 1508008 |  |
| ROH49 | 44 | 96 | 0.056518514 | 0.8120857 | 17 | 4508276 | 5736703 | 1228427 |  |
| ROH50 | 68 | 171 | 0.494251285 | 0.482037 | 17 | 22242355 | 30004501 | 7762146 |  |
| ROH51 | 5 | 25 | 2.723319923 | 0.09889213 | 17 | 30064220 | 31319575 | 1255355 |  |
| ROH52 | 8 | 26 | 0.785455943 | 0.3754777 | 17 | 33173925 | 36034365 | 2860440 |  |
| ROH53 | 109 | 229 | 0.503418939 | 0.4780017 | 17 | 36043653 | 47507019 | 11463366 |  |
| ROH54 | 312 | 617 | 4.722716352 | 0.02976669 | 17 | 49161581 | 53076986 | 3915405 |  |
| ROH55 | 172 | 396 | 0.015391249 | 0.9012666 | 17 | 55847256 | 64716397 | 8869141 |  |
| ROH56 | 144 | 275 | 3.111857754 | 0.07772426 | 17 | 66817866 | 67992704 | 1174838 |  |
| ROH57 | 1055 | 2486 | 1.636334902 | 0.2008291 | 17 | 68103004 | 69386951 | 1283947 |  |
| ROH58 | 22 | 28 | 4.319072839 | 0.03768745 | 17 | 72780491 | 74313086 | 1532595 |  |
| ROH59 | 32 | 66 | 0.21467946 | 0.6431242 | 17 | 79375998 | 80685533 | 1309535 |  |
| ROH60 | 371 | 840 | 0.01043472 | 0.9186372 | 18 | 13478189 | 14734475 | 1256286 |  |
| ROH61 | 367 | 924 | 3.197808162 | 0.07373703 | 18 | 15082789 | 22012716 | 6929927 |  |
| ROH62 | 6 | 20 | 0.68027913 | 0.4094906 | 18 | 22921715 | 24136254 | 1214539 |  |
| ROH63 | 47 | 114 | 0.135129455 | 0.7131718 | 18 | 24830305 | 45578935 | 20748630 |  |
| ROH64 | 31 | 68 | 0.030362636 | 0.86167 | 18 | 46368541 | 48233078 | 1864537 |  |
| ROH65 | 3 | 10 | 0.339547305 | 0.5600906 | 18 | 48404784 | 55209401 | 6804617 |  |
| ROH66 | 39 | 88 | 0.002354261 | 0.9613012 | 18 | 57687573 | 59048700 | 1361127 | Centromeric |
| ROH67 | 31 | 77 | 0.169023491 | 0.6809811 | 18 | 59416401 | 60780199 | 1363798 |  |
| ROH68 | 5 | 9 | 0.179032584 | 0.6722059 | 18 | 62559337 | 64877872 | 2318535 |  |
| ROH69 | 9 | 11 | 1.985283247 | 0.1588349 | 18 | 66234749 | 67251918 | 1017169 |  |
| ROH70 | 3 | 8 | 0.054522104 | 0.8153734 | 19 | 9119242 | 13267185 | 4147943 | Centromeric |
| ROH71 | 9 | 24 | 0.164049587 | 0.6854552 | 19 | 18512932 | 30149581 | 11636649 |  |
| ROH72 | 4 | 9 | 0.000406091 | 0.9839224 | 19 | 31304355 | 33702245 | 2397890 |  |
| ROH73 | 4 | 8 | 0.045120633 | 0.8317824 | 19 | 34442546 | 35482114 | 1039568 |  |
| ROH74 | 3 | 8 | 0.054522104 | 0.8153734 | 19 | 36078082 | 40004640 | 3926558 |  |
| ROH75 | 6 | 26 | 2.097010681 | 0.1475874 | 19 | 40009466 | 41008049 | 998583 |  |
| ROH76 | 10 | 26 | 0.127560521 | 0.7209749 | 19 | 41622615 | 45242173 | 3619558 |  |
| ROH77 | 46 | 103 | 0.00931263 | 0.9231219 | 19 | 47287939 | 48124652 | 836713 |  |
| ROH78 | 20 | 74 | 3.829665777 | 0.05035297 | 19 | 57828896 | 58518534 | 689638 |  |
| ROH79 | 32 | 68 | 0.105830756 | 0.7449416 | 20 | 7545361 | 8695689 | 1150328 |  |
| ROH80 | 3 | 16 | 1.947366196 | 0.162871 | 20 | 13276381 | 15143806 | 1867425 |  |
| ROH81 | 973 | 2167 | 0.576345732 | 0.4477483 | 20 | 18066079 | 18964360 | 898281 |  |
| ROH82 | 72 | 204 | 2.646748836 | 0.1037619 | 20 | 20382912 | 22807870 | 2424958 |  |
| ROH83 | 4 | 7 | 0.177905901 | 0.6731791 | 20 | 23344590 | 36527215 | 13182625 |  |
| ROH84 | 5 | 5 | 1.793617546 | 0.180486 | 20 | 36953097 | 38874083 | 1920986 |  |
| ROH85 | 19 | 40 | 0.080231727 | 0.7769836 | 20 | 38994419 | 40884676 | 1890257 |  |
| ROH86 | 796 | 1702 | 3.240300189 | 0.07184747 | 20 | 43335028 | 44949747 | 1614719 |  |
| ROH87 | 85 | 180 | 0.316705908 | 0.573594 | 20 | 46895949 | 48884531 | 1988582 |  |
| ROH88 | 48 | 106 | 0.031985259 | 0.85806 | 20 | 62912463 | 15862491 |  | Removed from analysis |
| ROH89 | 187 | 382 | 1.607619132 | 0.2048268 | 21 | 20937390 | 21721833 | 784443 |  |
| ROH90 | 157 | 342 | 0.22798634 | 0.633021 | 21 | 25962037 | 27795426 | 1833389 |  |
| ROH91 | 37 | 79 | 0.10651744 | 0.7441444 | 21 | 28862137 | 32638550 | 3776413 |  |
| ROH92 | 28 | 62 | 0.01541658 | 0.9011858 | 21 | 33249520 | 36119111 | 2869591 |  |
| ROH93 | 4 | 16 | 1.045178924 | 0.30662 | 21 | 36851619 | 37895398 | 1043779 |  |
| ROH94 | 503 | 1088 | 1.160115553 | 0.2814416 | 21 | 47015661 | 47577630 | 561969 | Centromeric |
| ROH95 | 6 | 11 | 0.183771504 | 0.6681519 | 22 | 20800835 | 23803722 | 3002887 |  |
| ROH96 | 8 | 12 | 0.851435548 | 0.3561465 | 22 | 23966720 | 25393476 | 1426756 |  |
| ROH97 | 96 | 198 | 0.661607137 | 0.4159931 | 22 | 28000527 | 32925170 | 4924643 |  |
| ROH98 | 5 | 11 | 0.00411778 | 0.9488349 | 22 | 34293516 | 35545811 | 1252295 |  |
| ROH99 | 8 | 9 | 2.200289933 | 0.1379848 | 22 | 37977305 | 39219932 | 1242627 |  |
| ROH100 | 31 | 88 | 1.135346175 | 0.2866379 | 1 | 7575666 | 9067142 | 1491476 |  |
| ROH101 | 81 | 181 | 0.020893876 | 0.8850684 | 1 | 9415727 | 10929928 | 1514201 |  |
| ROH102 | 14 | 24 | 0.721809514 | 0.395551 | 1 | 12278932 | 13920193 | 1641261 |  |
| ROH103 | 13 | 25 | 0.246404337 | 0.6196184 | 1 | 15643218 | 17554853 | 1911635 |  |
| ROH104 | 63 | 107 | 3.516423156 | 0.06076345 | 1 | 20735361 | 22491984 | 1756623 |  |
| ROH105 | 168 | 407 | 0.492463222 | 0.4828306 | 1 | 23171706 | 24881126 | 1709420 |  |
| ROH106 | 28 | 44 | 2.406065682 | 0.1208658 | 1 | 25333062 | 29827798 | 4494736 |  |
| ROH107 | 130 | 319 | 0.547486857 | 0.4593464 | 1 | 31315286 | 34177600 | 2862314 |  |
| ROH108 | 255 | 565 | 0.148888849 | 0.6995995 | 1 | 34797348 | 37384630 | 2587282 |  |
| ROH109 | 288 | 654 | 0.001900628 | 0.9652263 | 1 | 37538771 | 38559049 | 1020278 |  |
| ROH110 | 280 | 594 | 1.084833182 | 0.2976189 | 1 | 41821570 | 48196590 | 6375020 |  |
| ROH111 | 392 | 847 | 0.888527036 | 0.3458768 | 1 | 48359634 | 54787049 | 6427415 |  |
| ROH112 | 283 | 605 | 0.875498976 | 0.3494374 | 1 | 55474325 | 57068107 | 1593782 |  |
| ROH113 | 86 | 217 | 0.675275131 | 0.4112185 | 1 | 57975197 | 59277926 | 1302729 | Centromeric |
| ROH114 | 76 | 160 | 0.328769525 | 0.5663845 | 1 | 59985298 | 61629453 | 1644155 |  |
| ROH115 | 4 | 14 | 0.584835895 | 0.444423 | 1 | 63832640 | 68031411 | 4198771 |  |
| ROH116 | 7 | 8 | 1.850003148 | 0.173783 | 1 | 68581861 | 87921397 | 19339536 |  |
| ROH117 | 12 | 9 | 7.044110642 | 0.00795263 | 1 | 88251957 | 90492517 | 2240560 |  |
| ROH118 | 37 | 73 | 0.513974493 | 0.4734234 | 1 | 90600934 | 110132098 | 19531164 |  |
| ROH119 | 23 | 42 | 0.734225321 | 0.391517 | 1 | 112687359 | 156848106 | 44160747 |  |
| ROH120 | 189 | 400 | 0.749156445 | 0.3867434 | 1 | 160648549 | 162165433 | 1516884 |  |
| ROH121 | 4 | 9 | 0.000406091 | 0.9839224 | 1 | 162450093 | 164479479 | 2029386 |  |
| ROH122 | 76 | 189 | 0.435299654 | 0.5093999 | 1 | 165736880 | 167156816 | 1419936 |  |
| ROH123 | 215 | 510 | 0.278053263 | 0.59798 | 1 | 168407955 | 200037727 | 31629772 |  |
| ROH124 | 39 | 87 | 0.011717283 | 0.9138002 | 1 | 204322935 | 208410160 | 4087225 |  |
| ROH125 | 206 | 434 | 0.926587316 | 0.3357513 | 1 | 209269627 | 213594118 | 4324491 |  |
| ROH126 | 3 | 10 | 0.339547305 | 0.5600906 | 1 | 214504604 | 216229053 | 1724449 |  |
| ROH127 | 24 | 51 | 0.07910506 | 0.7785139 | 1 | 217620430 | 229144461 | 11524031 |  |
| ROH128 | 120 | 281 | 0.068840542 | 0.7930325 | 1 | 230817384 | 231912592 | 1095208 |  |
| ROH129 | 8 | 14 | 0.356336669 | 0.5505478 | 1 | 235068131 | 236220574 | 1152443 | Centromeric |
| ROH130 | 25 | 46 | 0.746554276 | 0.3875694 | 1 | 245969011 | 247203606 | 1234595 |  |
| ROH131 | 579 | 1341 | 0.154277316 | 0.6944809 | 1 | 247352621 | 1685800 |  | Removed from analysis |
| ROH132 | 90 | 179 | 1.14154664 | 0.2853258 | 2 | 8719699 | 9758621 | 1038922 |  |
| ROH133 | 373 | 967 | 5.514833337 | 0.01885587 | 2 | 13070276 | 15850849 | 2780573 |  |
| ROH134 | 23 | 60 | 0.310634291 | 0.5772912 | 2 | 16784998 | 20449174 | 3664176 |  |
| ROH135 | 250 | 488 | 4.368270594 | 0.03661413 | 2 | 20818764 | 23252410 | 2433646 | Centromeric |
| ROH136 | 111 | 274 | 0.540918621 | 0.4620523 | 2 | 23334779 | 30002690 | 6667911 |  |
| ROH137 | 8 | 17 | 0.026191479 | 0.8714336 | 2 | 31342300 | 33679231 | 2336931 |  |
| ROH138 | 77 | 187 | 0.233643958 | 0.628835 | 2 | 34203838 | 37634473 | 3430635 |  |
| ROH139 | 49 | 117 | 0.079117439 | 0.778497 | 2 | 38025672 | 45253128 | 7227456 |  |
| ROH140 | 11 | 15 | 1.712158382 | 0.1907056 | 2 | 66450555 | 66954363 | 503808 |  |
| ROH141 | 20 | 46 | 0.001372766 | 0.9704445 | 2 | 66972843 | 67687629 | 714786 |  |
| ROH142 | 5 | 20 | 1.307350013 | 0.252875 | 2 | 68998919 | 70842774 | 1843855 |  |
| ROH143 | 3 | 7 | 0.001238541 | 0.9719259 | 2 | 71866842 | 75037843 | 3171001 |  |
| ROH144 | 8 | 9 | 2.200289933 | 0.1379848 | 2 | 75681330 | 77377286 | 1695956 |  |
| ROH145 | 6 | 16 | 0.109205062 | 0.741051 | 2 | 77691733 | 79225690 | 1533957 |  |
| ROH146 | 5 | 13 | 0.063626388 | 0.8008535 | 2 | 80217014 | 87091247 | 6874233 |  |
| ROH147 | 35 | 72 | 0.247392842 | 0.6189169 | 2 | 89125131 | 106024451 | 16899320 |  |
| ROH148 | 5 | 14 | 0.158202145 | 0.6908172 | 2 | 106071159 | 121225558 | 15154399 |  |
| ROH149 | 5 | 9 | 0.179032584 | 0.6722059 | 2 | 121916540 | 133759852 | 11843312 |  |
| ROH150 | 921 | 2081 | 0.066318939 | 0.7967738 | 2 | 171548493 | 174251944 | 2703451 |  |
| ROH151 | 29 | 39 | 4.766185236 | 0.02902407 | 2 | 174554115 | 177113353 | 2559238 |  |
| ROH152 | 6 | 21 | 0.878313328 | 0.3486641 | 2 | 177330967 | 192364713 | 15033746 | Centromeric |
| ROH153 | 166 | 354 | 0.524756992 | 0.4688195 | 2 | 193052353 | 206554544 | 13502191 |  |
| ROH154 | 681 | 1504 | 0.624514798 | 0.4293745 | 2 | 206743798 | 208049581 | 1305783 |  |
| ROH155 | 13 | 11 | 6.354125812 | 0.01171086 | 2 | 208386024 | 211969338 | 3583314 |  |
| ROH156 | 178 | 384 | 0.385983788 | 0.5344181 | 2 | 212435213 | 216139373 | 3704160 |  |
| ROH157 | 332 | 806 | 1.133524976 | 0.2870247 | 2 | 217506265 | 217974444 | 468179 |  |
| ROH158 | 57 | 130 | 8.59096E-05 | 0.9926047 | 2 | 218712306 | 220599533 | 1887227 |  |
| ROH159 | 32 | 52 | 2.304099436 | 0.129033 | 2 | 223338071 | 227922470 | 4584399 |  |
| ROH160 | 16 | 50 | 1.234532186 | 0.2665274 | 2 | 232176541 | 233387635 | 1211094 |  |
| ROH161 | 163 | 339 | 0.973040751 | 0.323923 | 2 | 238833264 | 239638300 | 805036 |  |
| ROH162 | 9 | 33 | 1.643701597 | 0.1998183 | 2 | 241506669 | 242824974 | 1318305 |  |
| ROH163 | 8 | 9 | 2.200289933 | 0.1379848 | 3 | 104972 | 1164339 | 1059367 |  |
| ROH164 | 18 | 25 | 2.626986709 | 0.1050609 | 3 | 3755608 | 4609648 | 854040 | Centromeric |
| ROH165 | 17 | 23 | 2.725799193 | 0.09873869 | 3 | 9416832 | 10383517 | 966685 |  |
| ROH166 | 7 | 6 | 3.344244184 | 0.06744044 | 3 | 11628813 | 13057791 | 1428978 |  |
| ROH167 | 5 | 8 | 0.388141833 | 0.5332778 | 3 | 14807375 | 16364919 | 1557544 |  |
| ROH168 | 17 | 51 | 0.983396378 | 0.3213617 | 3 | 16516102 | 21500623 | 4984521 |  |
| ROH169 | 8 | 7 | 3.691986234 | 0.05467447 | 3 | 22162351 | 23985809 | 1823458 |  |
| ROH170 | 10 | 30 | 0.576291133 | 0.4477698 | 3 | 30668684 | 31738265 | 1069581 |  |
| ROH171 | 11 | 22 | 0.124431689 | 0.7242768 | 3 | 32578505 | 54077726 | 21499221 |  |
| ROH172 | 8 | 10 | 1.651908848 | 0.1986993 | 3 | 55571760 | 59539911 | 3968151 | Centromeric |
| ROH173 | 7 | 22 | 0.557893351 | 0.4551103 | 3 | 61014080 | 61549295 | 535215 | Centromeric |
| ROH174 | 4 | 18 | 1.582219836 | 0.2084412 | 3 | 65068148 | 65504146 | 435998 |  |
| ROH175 | 5 | 19 | 1.06381455 | 0.3023468 | 3 | 66835382 | 70836444 | 4001062 |  |
| ROH176 | 47 | 104 | 0.027378179 | 0.8685792 | 3 | 74266309 | 116682766 | 42416457 | Centromeric |
| ROH177 | 7 | 15 | 0.017752568 | 0.8940046 | 3 | 117077219 | 122780413 | 5703194 |  |
| ROH178 | 42 | 99 | 0.035613915 | 0.850315 | 3 | 122783953 | 126220539 | 3436586 |  |
| ROH179 | 33 | 60 | 1.097887954 | 0.2947301 | 3 | 126945750 | 133167363 | 6221613 |  |
| ROH180 | 20 | 44 | 0.016577744 | 0.8975518 | 3 | 139807568 | 142901537 | 3093969 |  |
| ROH181 | 305 | 742 | 1.095488406 | 0.2952583 | 3 | 143405663 | 147390505 | 3984842 |  |
| ROH182 | 6 | 16 | 0.109205062 | 0.741051 | 3 | 147481380 | 148903923 | 1422543 |  |
| ROH183 | 354 | 750 | 1.47353137 | 0.2247893 | 3 | 150732589 | 170874398 | 20141809 |  |
| ROH184 | 368 | 774 | 1.863315709 | 0.1722426 | 3 | 173322248 | 174354964 | 1032716 |  |
| ROH185 | 523 | 1083 | 4.066715875 | 0.04373623 | 3 | 175206683 | 176787698 | 1581015 |  |
| ROH186 | 35 | 115 | 3.72056179 | 0.05374628 | 3 | 177254210 | 185926701 | 8672491 |  |
| ROH187 | 20 | 28 | 2.835055616 | 0.09222761 | 3 | 190978515 | 192038154 | 1059639 |  |
| ROH188 | 37 | 87 | 0.02696719 | 0.8695604 | 3 | 194733941 | 195777211 | 1043270 |  |
| ROH189 | 8 | 6 | 4.691670209 | 0.03030917 | 3 | 197833758 | 3652592 |  | Removed from analysis |
| ROH190 | 4 | 7 | 0.177905901 | 0.6731791 | 4 | 11513562 | 13755011 | 2241449 |  |
| ROH191 | 5 | 12 | 0.009729857 | 0.9214241 | 4 | 13787277 | 16410294 | 2623017 |  |
| ROH192 | 35 | 69 | 0.490993327 | 0.4834845 | 4 | 17196209 | 23853011 | 6656802 |  |
| ROH193 | 13 | 36 | 0.368774088 | 0.5436733 | 4 | 26197873 | 37565263 | 11367390 |  |
| ROH194 | 18 | 22 | 3.981241495 | 0.04600964 | 4 | 38394714 | 40397913 | 2003199 |  |
| ROH195 | 12 | 22 | 0.368382259 | 0.5438874 | 4 | 41548580 | 57103047 | 15554467 |  |
| ROH196 | 15 | 28 | 0.389830847 | 0.5323883 | 4 | 57680805 | 77724484 | 20043679 |  |
| ROH197 | 646 | 1391 | 1.905143921 | 0.1675037 | 4 | 77854979 | 96170439 | 18315460 |  |
| ROH198 | 229 | 568 | 1.3321042 | 0.2484312 | 4 | 96374272 | 113989119 | 17614847 |  |
| ROH199 | 5 | 7 | 0.705346802 | 0.4009929 | 4 | 114139428 | 139496147 | 25356719 |  |
| ROH200 | 54 | 106 | 0.808457501 | 0.3685767 | 4 | 169726027 | 174631819 | 4905792 |  |
| ROH201 | 491 | 1126 | 0.021091298 | 0.8845305 | 4 | 175928058 | 177184716 | 1256658 |  |
| ROH202 | 11 | 19 | 0.538121577 | 0.4632123 | 4 | 178283967 | 179399523 | 1115556 |  |
| ROH203 | 80 | 191 | 0.130457678 | 0.717958 | 4 | 190903688 | 1186086 | -189717602 | Removed from analysis |
| ROH204 | 6 | 10 | 0.36928535 | 0.5433941 | 5 | 11428523 | 13760721 | 2332198 |  |
| ROH205 | 324 | 806 | 2.12447234 | 0.1449627 | 5 | 14707600 | 16528257 | 1820657 |  |
| ROH206 | 6 | 6 | 2.152917549 | 0.1422993 | 5 | 17400274 | 31609654 | 14209380 |  |
| ROH207 | 24 | 28 | 6.042582647 | 0.01396484 | 5 | 32663496 | 33703734 | 1040238 |  |
| ROH208 | 11 | 26 | 0.010734939 | 0.9174792 | 5 | 34772786 | 38112559 | 3339773 |  |
| ROH209 | 27 | 43 | 2.164703168 | 0.1412119 | 5 | 38709629 | 53594702 | 14885073 |  |
| ROH210 | 11 | 36 | 1.127016631 | 0.2884126 | 5 | 56730383 | 65873081 | 9142698 |  |
| ROH211 | 83 | 161 | 1.460879281 | 0.2267903 | 5 | 67755091 | 68747990 | 992899 | Centromeric |
| ROH212 | 3 | 13 | 1.046257439 | 0.3063706 | 5 | 71200910 | 73069226 | 1868316 |  |
| ROH213 | 15 | 44 | 0.726219677 | 0.3941113 | 5 | 73879195 | 75642486 | 1763291 |  |
| ROH214 | 16 | 30 | 0.398120968 | 0.5280613 | 5 | 77645784 | 79092370 | 1446586 |  |
| ROH215 | 26 | 25 | 10.14667418 | 0.001445618 | 5 | 79228649 | 82053933 | 2825284 |  |
| ROH216 | 52 | 147 | 1.851054139 | 0.1736608 | 5 | 82980449 | 115789599 | 32809150 |  |
| ROH217 | 94 | 192 | 0.777878841 | 0.3777907 | 5 | 116228312 | 123741332 | 7513020 |  |
| ROH218 | 6 | 6 | 2.152917549 | 0.1422993 | 5 | 124512345 | 132612643 | 8100298 |  |
| ROH219 | 31 | 38 | 6.825723177 | 0.008985417 | 5 | 133051079 | 134639915 | 1588836 |  |
| ROH220 | 43 | 112 | 0.572871083 | 0.4491203 | 5 | 142228026 | 146561416 | 4333390 |  |
| ROH221 | 5 | 25 | 2.723319923 | 0.09889213 | 5 | 146675710 | 148206440 | 1530730 |  |
| ROH222 | 23 | 70 | 1.484326596 | 0.2230988 | 5 | 150971110 | 158948962 | 7977852 |  |
| ROH223 | 13 | 20 | 1.233272163 | 0.2667716 | 5 | 159865579 | 162086807 | 2221228 |  |
| ROH224 | 9 | 12 | 1.51371573 | 0.218573 | 5 | 162095410 | 163511740 | 1416330 |  |
| ROH225 | 200 | 444 | 0.098285197 | 0.7538973 | 5 | 163586269 | 165029143 | 1442874 |  |
| ROH226 | 35 | 109 | 2.667622874 | 0.1024089 | 5 | 175244097 | 177260129 | 2016032 |  |
| ROH227 | 51 | 108 | 0.187271838 | 0.665197 | 6 | 2374821 | 2693442 | 318621 |  |
| ROH228 | 11 | 17 | 1.020381866 | 0.3124284 | 6 | 7815519 | 9181572 | 1366053 |  |
| ROH229 | 13 | 24 | 0.374825032 | 0.5403859 | 6 | 12366819 | 13623324 | 1256505 |  |
| ROH230 | 9 | 20 | 0.003746974 | 0.9511899 | 6 | 17181845 | 19643688 | 2461843 |  |
| ROH231 | 6 | 25 | 1.827677305 | 0.1764021 | 6 | 20204711 | 21462277 | 1257566 |  |
| ROH232 | 290 | 698 | 0.722319061 | 0.3953843 | 6 | 22155662 | 23380783 | 1225121 |  |
| ROH233 | 11 | 23 | 0.054589465 | 0.8152615 | 6 | 23656880 | 37177783 | 13520903 |  |
| ROH234 | 12 | 29 | 0.030081295 | 0.8623059 | 6 | 37585221 | 40344459 | 2759238 |  |
| ROH235 | 130 | 290 | 0.04068432 | 0.8401485 | 6 | 42369308 | 43764359 | 1395051 |  |
| ROH236 | 7 | 20 | 0.268815517 | 0.6041274 | 6 | 44324600 | 53088181 | 8763581 |  |
| ROH237 | 23 | 57 | 0.118424231 | 0.7307499 | 6 | 53398559 | 88780388 | 35381829 |  |
| ROH238 | 83 | 153 | 2.493108363 | 0.1143457 | 6 | 88884821 | 90674451 | 1789630 |  |
| ROH239 | 124 | 275 | 0.063593245 | 0.8009042 | 6 | 91802492 | 106252999 | 14450507 |  |
| ROH240 | 318 | 791 | 2.073792586 | 0.1498485 | 6 | 125385470 | 130031215 | 4645745 |  |
| ROH241 | 605 | 1418 | 0.480350614 | 0.4882635 | 6 | 130236932 | 134317730 | 4080798 |  |
| ROH242 | 5 | 7 | 0.705346802 | 0.4009929 | 6 | 134938220 | 137607358 | 2669138 |  |
| ROH243 | 30 | 53 | 1.255863402 | 0.2624355 | 6 | 139683605 | 148160266 | 8476661 |  |
| ROH244 | 126 | 198 | 11.20863428 | 0.000814176 | 6 | 149237490 | 150525260 | 1287770 |  |
| ROH245 | 103 | 228 | 0.05993156 | 0.8066041 | 6 | 152565897 | 153704558 | 1138661 |  |
| ROH246 | 132 | 264 | 1.569704962 | 0.2102499 | 6 | 156763643 | 159539213 | 2775570 |  |
| ROH247 | 332 | 696 | 1.788268143 | 0.1811373 | 6 | 159895128 | 161772739 | 1877611 |  |
| ROH248 | 41 | 70 | 2.193611679 | 0.138584 | 6 | 167269979 | 168317994 | 1048015 |  |
| ROH249 | 10 | 19 | 0.216485567 | 0.6417309 | 6 | 169727601 | 170737157 | 1009556 |  |
| ROH250 | 5 | 6 | 1.160096042 | 0.2814456 | 7 | 3276268 | 4270682 | 994414 |  |
| ROH251 | 8 | 6 | 4.691670209 | 0.03030917 | 7 | 5375542 | 7052722 | 1677180 |  |
| ROH252 | 5 | 10 | 0.05642346 | 0.8122408 | 7 | 9622923 | 11232243 | 1609320 |  |
| ROH253 | 3 | 10 | 0.339547305 | 0.5600906 | 7 | 11665731 | 12725678 | 1059947 |  |
| ROH254 | 31 | 78 | 0.223918029 | 0.6360707 | 7 | 14951097 | 16676955 | 1725858 |  |
| ROH255 | 46 | 108 | 0.030563685 | 0.8612174 | 7 | 17540774 | 18861140 | 1320366 |  |
| ROH256 | 122 | 273 | 0.027508497 | 0.8682696 | 7 | 19019671 | 20139644 | 1119973 |  |
| ROH257 | 92 | 180 | 1.45984748 | 0.2269544 | 7 | 22512880 | 28338080 | 5825200 |  |
| ROH258 | 830 | 1875 | 0.059004485 | 0.8080764 | 7 | 29540205 | 30809567 | 1269362 |  |
| ROH259 | 247 | 565 | 0.003746347 | 0.951194 | 7 | 32030880 | 36345194 | 4314314 |  |
| ROH260 | 120 | 261 | 0.183319333 | 0.668536 | 7 | 42209334 | 47494033 | 5284699 | Centromeric |
| ROH261 | 43 | 77 | 1.628730399 | 0.2018787 | 7 | 47687938 | 78068055 | 30380117 |  |
| ROH262 | 267 | 573 | 0.723959597 | 0.3948482 | 7 | 78403188 | 79799613 | 1396425 |  |
| ROH263 | 191 | 429 | 0.027626404 | 0.8679902 | 7 | 82227551 | 93322256 | 11094705 |  |
| ROH264 | 13 | 24 | 0.374825032 | 0.5403859 | 7 | 93407398 | 105450966 | 12043568 |  |
| ROH265 | 7 | 16 | 6.50496E-05 | 0.9935649 | 7 | 106349163 | 130582724 | 24233561 |  |
| ROH266 | 10 | 18 | 0.358737951 | 0.549208 | 7 | 131889849 | 137496871 | 5607022 |  |
| ROH267 | 3 | 15 | 1.631363901 | 0.2015145 | 7 | 139688004 | 141729747 | 2041743 |  |
| ROH268 | 6 | 13 | 0.01022542 | 0.9194546 | 7 | 141816117 | 147469901 | 5653784 |  |
| ROH269 | 6 | 24 | 1.569873056 | 0.2102255 | 7 | 149563894 | 150490130 | 926236 |  |
| ROH270 | 13 | 24 | 0.374825032 | 0.5403859 | 7 | 157934557 | 159089523 | 1154966 |  |
| ROH271 | 53 | 117 | 0.036213732 | 0.8490748 | 8 | 8695574 | 11076635 | 2381061 |  |
| ROH272 | 20 | 46 | 0.001372766 | 0.9704445 | 8 | 11330364 | 12952230 | 1621866 |  |
| ROH273 | 22 | 46 | 0.109679819 | 0.740509 | 8 | 13898663 | 16691422 | 2792759 |  |
| ROH274 | 8 | 14 | 0.356336669 | 0.5505478 | 8 | 23528230 | 25193338 | 1665108 |  |
| ROH275 | 496 | 1144 | 0.071265451 | 0.7895031 | 8 | 26267362 | 29367054 | 3099692 |  |
| ROH276 | 14 | 39 | 0.422587271 | 0.5156489 | 8 | 29491696 | 58929688 | 29437992 | Centromeric |
| ROH277 | 171 | 422 | 0.852962237 | 0.3557157 | 8 | 59599500 | 69057145 | 9457645 |  |
| ROH278 | 129 | 292 | 0.003513879 | 0.9527307 | 8 | 69371911 | 70373110 | 1001199 |  |
| ROH279 | 255 | 630 | 1.365868078 | 0.2425231 | 8 | 70564677 | 72245087 | 1680410 |  |
| ROH280 | 5 | 9 | 0.179032584 | 0.6722059 | 8 | 96896725 | 98796043 | 1899318 |  |
| ROH281 | 5 | 5 | 1.793617546 | 0.180486 | 8 | 99150174 | 101531518 | 2381344 |  |
| ROH282 | 41 | 79 | 0.768238969 | 0.3807624 | 8 | 102489035 | 103572247 | 1083212 |  |
| ROH283 | 20 | 24 | 4.660980359 | 0.03085557 | 8 | 103782719 | 122330471 | 18547752 |  |
| ROH284 | 20 | 57 | 0.755696036 | 0.3846788 | 8 | 122438189 | 122955670 | 517481 |  |
| ROH285 | 455 | 1052 | 0.090959181 | 0.7629613 | 8 | 129155329 | 131640843 | 2485514 |  |
| ROH286 | 133 | 320 | 0.301971668 | 0.582649 | 8 | 136203934 | 138796025 | 2592091 |  |
| ROH287 | 14 | 27 | 0.256813206 | 0.612318 | 8 | 143761931 | 146271129 | 2509198 |  |
| ROH288 | 5 | 7 | 0.705346802 | 0.4009929 | 9 | 5385783 | 6849317 | 1463534 |  |
| ROH289 | 9 | 30 | 1.022201685 | 0.3119973 | 9 | 10602787 | 12908447 | 2305660 |  |
| ROH290 | 14 | 31 | 0.00766163 | 0.9302497 | 9 | 20289153 | 23127562 | 2838409 |  |
| ROH291 | 27 | 59 | 0.032011468 | 0.8580025 | 9 | 23569119 | 25279649 | 1710530 |  |
| ROH292 | 17 | 21 | 3.64504844 | 0.05623617 | 9 | 25775262 | 27338480 | 1563218 |  |
| ROH293 | 392 | 952 | 1.398851667 | 0.2369159 | 9 | 28252086 | 32725773 | 4473687 |  |
| ROH294 | 13 | 19 | 1.550180415 | 0.2131088 | 9 | 32911250 | 36727013 | 3815763 |  |
| ROH295 | 171 | 419 | 0.706319626 | 0.4006683 | 9 | 37046091 | 37684753 | 638662 |  |
| ROH296 | 18 | 13 | 11.14560453 | 0.00084231 | 9 | 71114312 | 74260812 | 3146500 |  |
| ROH297 | 129 | 301 | 0.05643047 | 0.8122294 | 9 | 74300640 | 77684690 | 3384050 |  |
| ROH298 | 11 | 30 | 0.263726519 | 0.6075715 | 9 | 79677419 | 81301082 | 1623663 |  |
| ROH299 | 4 | 12 | 0.229775143 | 0.6316906 | 9 | 82455838 | 87221357 | 4765519 |  |
| ROH300 | 5 | 8 | 0.388141833 | 0.5332778 | 9 | 93658952 | 100904179 | 7245227 |  |
| ROH301 | 13 | 24 | 0.374825032 | 0.5403859 | 9 | 101680380 | 104449098 | 2768718 |  |
| ROH302 | 27 | 56 | 0.161210875 | 0.6880441 | 9 | 104943443 | 109470258 | 4526815 | Centromeric |
| ROH303 | 33 | 79 | 0.058878763 | 0.808277 | 9 | 110875895 | 112081994 | 1206099 |  |
| ROH304 | 314 | 683 | 0.523612451 | 0.4693048 | 9 | 113512773 | 115777556 | 2264783 |  |
| ROH305 | 26 | 51 | 0.388574721 | 0.5330495 | 9 | 117665435 | 119011391 | 1345956 |  |
| ROH306 | 17 | 43 | 0.135422175 | 0.7128751 | 9 | 119128575 | 121524247 | 2395672 |  |
| ROH307 | 429 | 1010 | 0.411479438 | 0.5212196 | 9 | 122596036 | 132111298 | 9515262 |  |
| ROH308 | 79 | 138 | 3.661018331 | 0.05569958 | 9 | 141066491 | 1479478 |  | Removed from analysis |
| ROH309 | 6 | 10 | 0.36928535 | 0.5433941 | 10 | 8757444 | 10540164 | 1782720 |  |
| ROH310 | 9 | 15 | 0.554522447 | 0.4564757 | 10 | 24667091 | 26704514 | 2037423 |  |
| ROH311 | 6 | 6 | 2.152917549 | 0.1422993 | 10 | 26746468 | 29075122 | 2328654 |  |
| ROH312 | 64 | 145 | 0.001225013 | 0.9720796 | 10 | 30816383 | 33770092 | 2953709 |  |
| ROH313 | 10 | 8 | 5.337356309 | 0.0208731 | 10 | 34189978 | 53425328 | 19235350 |  |
| ROH314 | 59 | 103 | 2.725675124 | 0.09874636 | 10 | 54832274 | 71119208 | 16286934 |  |
| ROH315 | 29 | 33 | 7.797915667 | 0.005230654 | 10 | 73414577 | 78005230 | 4590653 |  |
| ROH316 | 5 | 7 | 0.705346802 | 0.4009929 | 10 | 78163768 | 78694965 | 531197 |  |
| ROH317 | 746 | 1777 | 1.600692952 | 0.205805 | 10 | 81173663 | 82474165 | 1300502 |  |
| ROH318 | 8 | 7 | 3.691986234 | 0.05467447 | 10 | 82479714 | 90904002 | 8424288 |  |
| ROH319 | 4 | 7 | 0.177905901 | 0.6731791 | 10 | 91263587 | 95378547 | 4114960 |  |
| ROH320 | 50 | 86 | 2.554169158 | 0.1100036 | 10 | 100036209 | 114342159 | 14305950 |  |
| ROH321 | 64 | 144 | 0.006671718 | 0.9349007 | 10 | 116046579 | 118981071 | 2934492 |  |
| ROH322 | 4 | 13 | 0.391833255 | 0.5313373 | 10 | 119641986 | 122068766 | 2426780 |  |
| ROH323 | 286 | 670 | 0.183505178 | 0.6683781 | 10 | 124851234 | 125319543 | 468309 |  |
| ROH324 | 4 | 7 | 0.177905901 | 0.6731791 | 10 | 134039891 | 135071004 | 1031113 |  |
| ROH325 | 173 | 415 | 0.357828319 | 0.5497148 | 11 | 715420 | 1779138 | 1063718 |  |
| ROH326 | 2 | 9 | 0.789944652 | 0.3741169 | 11 | 3715036 | 4687238 | 972202 |  |
| ROH327 | 3 | 10 | 0.339547305 | 0.5600906 | 11 | 8332942 | 10702517 | 2369575 |  |
| ROH328 | 6 | 8 | 1.008197536 | 0.315335 | 11 | 13206160 | 17458136 | 4251976 |  |
| ROH329 | 21 | 62 | 1.074900336 | 0.2998412 | 11 | 21581005 | 33696361 | 12115356 |  |
| ROH330 | 24 | 54 | 0.002457948 | 0.9604589 | 11 | 44994060 | 60830367 | 15836307 |  |
| ROH331 | 84 | 215 | 0.859181726 | 0.3539678 | 11 | 60835322 | 69078794 | 8243472 |  |
| ROH332 | 62 | 158 | 0.580530745 | 0.4461044 | 11 | 70645921 | 76261533 | 5615612 |  |
| ROH333 | 6 | 11 | 0.183771504 | 0.6681519 | 11 | 76455618 | 78818226 | 2362608 |  |
| ROH334 | 44 | 95 | 0.087206922 | 0.7677585 | 11 | 80690336 | 94774208 | 14083872 |  |
| ROH335 | 454 | 1028 | 0.013012434 | 0.9091807 | 11 | 95938418 | 113025146 | 17086728 | Centromeric |
| ROH336 | 29 | 52 | 1.080947986 | 0.2984856 | 11 | 114191016 | 116104358 | 1913342 |  |
| ROH337 | 11 | 41 | 2.162926971 | 0.1413752 | 11 | 116376840 | 117690543 | 1313703 |  |
| ROH338 | 10 | 21 | 0.044756367 | 0.8324527 | 11 | 117788313 | 119376672 | 1588359 |  |
| ROH339 | 154 | 355 | 0.016974287 | 0.8963406 | 11 | 123438030 | 124657183 | 1219153 |  |
| ROH340 | 166 | 456 | 4.679007188 | 0.03053339 | 12 | 10281255 | 11908529 | 1627274 |  |
| ROH341 | 5 | 13 | 0.063626388 | 0.8008535 | 12 | 13493381 | 16408940 | 2915559 |  |
| ROH342 | 11 | 25 | 3.22178E-05 | 0.9954712 | 12 | 16423287 | 20070323 | 3647036 |  |
| ROH343 | 308 | 756 | 1.432475521 | 0.2313609 | 12 | 20592201 | 22988849 | 2396648 |  |
| ROH344 | 58 | 141 | 0.180020427 | 0.6713557 | 12 | 26488505 | 29776054 | 3287549 |  |
| ROH345 | 396 | 940 | 0.582512622 | 0.4453291 | 12 | 29829680 | 30990587 | 1160907 |  |
| ROH346 | 7 | 11 | 0.597162864 | 0.4396626 | 12 | 31029524 | 51922480 | 20892956 |  |
| ROH347 | 8 | 10 | 1.651908848 | 0.1986993 | 12 | 53035673 | 54963907 | 1928234 |  |
| ROH348 | 6 | 24 | 1.569873056 | 0.2102255 | 12 | 54980215 | 66412964 | 11432749 |  |
| ROH349 | 8 | 15 | 0.198446429 | 0.6559778 | 12 | 68829232 | 76099541 | 7270309 |  |
| ROH350 | 6 | 8 | 1.008197536 | 0.315335 | 12 | 92822306 | 93673499 | 851193 |  |
| ROH351 | 7 | 12 | 0.359985562 | 0.5485143 | 12 | 95388843 | 96036307 | 647464 |  |
| ROH352 | 42 | 99 | 0.035613915 | 0.850315 | 12 | 96436653 | 101929013 | 5492360 |  |
| ROH353 | 7 | 27 | 1.586493055 | 0.2078279 | 12 | 101988525 | 104132365 | 2143840 |  |
| ROH354 | 227 | 543 | 0.430825997 | 0.5115839 | 12 | 105134621 | 108644189 | 3509568 |  |
| ROH355 | 10 | 27 | 0.213009116 | 0.644419 | 12 | 108668552 | 113916804 | 5248252 |  |
| ROH356 | 111 | 288 | 1.441218987 | 0.2299422 | 12 | 119763994 | 124854903 | 5090909 |  |
| ROH357 | 6 | 24 | 1.569873056 | 0.2102255 | 12 | 132663423 | 20969210 |  | Removed from analysis |
| ROH358 | 7 | 18 | 0.074659813 | 0.7846691 | 13 | 33730275 | 34696072 | 965797 |  |
| ROH359 | 32 | 59 | 0.940574742 | 0.3321301 | 13 | 35104243 | 36402079 | 1297836 |  |
| ROH360 | 6 | 9 | 0.63814889 | 0.4243819 | 13 | 40943836 | 43492349 | 2548513 |  |
| ROH361 | 5 | 11 | 0.00411778 | 0.9488349 | 13 | 44058279 | 46728924 | 2670645 |  |
| ROH362 | 34 | 87 | 0.337735097 | 0.5611395 | 13 | 47432610 | 71291350 | 23858740 |  |
| ROH363 | 76 | 186 | 0.289849221 | 0.5903172 | 13 | 71380349 | 72978869 | 1598520 |  |
| ROH364 | 12 | 20 | 0.74015753 | 0.3896104 | 13 | 75867907 | 91926396 | 16058489 |  |
| ROH365 | 4 | 8 | 0.045120633 | 0.8317824 | 13 | 92828701 | 94768636 | 1939935 |  |
| ROH366 | 318 | 712 | 0.073700452 | 0.7860232 | 13 | 95820852 | 98509302 | 2688450 |  |
| ROH367 | 7 | 10 | 0.913958196 | 0.3390664 | 13 | 99265701 | 100558473 | 1292772 |  |
| ROH368 | 47 | 85 | 1.644512598 | 0.1997074 | 13 | 101926339 | 103051897 | 1125558 |  |
| ROH369 | 3 | 11 | 0.545845447 | 0.4600202 | 14 | 24799843 | 25587842 | 787999 |  |
| ROH370 | 5 | 7 | 0.705346802 | 0.4009929 | 14 | 30807894 | 32661232 | 1853338 |  |
| ROH371 | 7 | 11 | 0.597162864 | 0.4396626 | 14 | 34777890 | 37190065 | 2412175 |  |
| ROH372 | 0 | 10 | 4.396873764 | 0.03600487 | 14 | 37277315 | 51417886 | 14140571 |  |
| ROH373 | 30 | 59 | 0.43386776 | 0.5100972 | 14 | 52373353 | 54310925 | 1937572 |  |
| ROH374 | 1 | 12 | 3.198460599 | 0.07370762 | 14 | 55083965 | 56507568 | 1423603 |  |
| ROH375 | 89 | 187 | 0.406531751 | 0.5237351 | 14 | 57075920 | 75790221 | 18714301 |  |
| ROH376 | 538 | 1198 | 0.244325561 | 0.6210993 | 14 | 77352149 | 89488741 | 12136592 |  |
| ROH377 | 35 | 78 | 0.011517852 | 0.9145341 | 14 | 91186670 | 92566983 | 1380313 |  |
| ROH378 | 22 | 57 | 0.267254989 | 0.6051791 | 14 | 93064069 | 94417675 | 1353606 |  |
| ROH379 | 9 | 20 | 0.003746974 | 0.9511899 | 14 | 100031142 | 101107363 | 1076221 | Centromeric |
| ROH380 | 15 | 31 | 0.095958859 | 0.7567341 | 15 | 24050258 | 25127067 | 1076809 |  |
| ROH381 | 8 | 11 | 1.207482572 | 0.2718313 | 15 | 27687150 | 29675431 | 1988281 |  |
| ROH382 | 2 | 10 | 1.086702028 | 0.2972031 | 15 | 29891949 | 32970550 | 3078601 |  |
| ROH383 | 12 | 17 | 1.621586316 | 0.2028707 | 15 | 37887501 | 38361447 | 473946 |  |
| ROH384 | 25 | 57 | 2.30823E-05 | 0.9961667 | 15 | 39497993 | 57746815 | 18248822 |  |
| ROH385 | 206 | 404 | 3.325868121 | 0.068198 | 15 | 58768822 | 60086249 | 1317427 |  |
| ROH386 | 6 | 20 | 0.68027913 | 0.4094906 | 15 | 61976593 | 62596003 | 619410 |  |
| ROH387 | 6 | 9 | 0.63814889 | 0.4243819 | 15 | 63286060 | 66420014 | 3133954 |  |
| ROH388 | 4 | 8 | 0.045120633 | 0.8317824 | 15 | 67192716 | 68758506 | 1565790 |  |
| ROH389 | 20 | 44 | 0.016577744 | 0.8975518 | 15 | 68974860 | 70071778 | 1096918 |  |
| ROH390 | 9 | 16 | 0.356307656 | 0.5505641 | 15 | 80086063 | 80563203 | 477140 |  |
| ROH391 | 312 | 689 | 0.235087701 | 0.6277768 | 15 | 81578139 | 87307845 | 5729706 |  |
| ROH392 | 7 | 12 | 0.359985562 | 0.5485143 | 15 | 90675264 | 91337479 | 662215 |  |
| ROH393 | 6 | 9 | 0.63814889 | 0.4243819 | 16 | 1624142 | 2512523 | 888381 |  |

**Supplementary Table 1: Details of the ROH found in the discovery dataset.** In the annotation column, “removed from analysis” indicates that the ROH spanned two chromosomes and was removed from the analysis, and “centromeric” indicates that the ROH spanned the centromere of a chromosome. Coordinates are from NCBI37.

| ROH ID | No. in Cases | No. in Controls | χ² | *P*-value | Chromosome | Start bp (NCBI37) | End bp (NCBI37) | Length bp | Annotation |
| --- | --- | --- | --- | --- | --- | --- | --- | --- | --- |
| ROH1 | 5 | 38 | 0.01581624 | 0.8999198 | 1 | 834753 | 2211849 | 1377096 |  |
| ROH2 | 4 | 37 | 0.068026764 | 0.7942318 | 1 | 6020992 | 7338813 | 1317821 |  |
| ROH3 | 2 | 35 | 1.197953936 | 0.273731 | 1 | 7600905 | 9075221 | 1474316 |  |
| ROH4 | 21 | 152 | 0.222038534 | 0.637491 | 1 | 9453902 | 10820215 | 1366313 |  |
| ROH5 | 2 | 11 | 0.251780719 | 0.615824 | 1 | 12206866 | 13913180 | 1706314 |  |
| ROH6 | 2 | 35 | 1.197953936 | 0.273731 | 1 | 15705031 | 17459855 | 1754824 |  |
| ROH7 | 4 | 74 | 2.794992629 | 0.0945592 | 1 | 20893965 | 24485941 | 3591976 |  |
| ROH8 | 46 | 309 | 1.409026489 | 0.2352181 | 1 | 25297184 | 29802337 | 4505153 |  |
| ROH9 | 15 | 177 | 2.074105929 | 0.1498177 | 1 | 31297519 | 34112263 | 2814744 |  |
| ROH10 | 235 | 1802 | 0.719393396 | 0.396343 | 1 | 34921075 | 38575606 | 3654531 |  |
| ROH11 | 237 | 1998 | 0.571294865 | 0.4497448 | 1 | 39222532 | 53787633 | 14565101 |  |
| ROH12 | 5 | 39 | 0.005056367 | 0.9433117 | 1 | 55504650 | 57057316 | 1552666 |  |
| ROH13 | 3 | 23 | 0.006906222 | 0.9337691 | 1 | 59733132 | 61629453 | 1896321 |  |
| ROH14 | 3 | 10 | 1.926273302 | 0.1651667 | 1 | 62797950 | 63872871 | 1074921 |  |
| ROH15 | 134 | 896 | 4.731257888 | 0.02961922 | 1 | 64295971 | 81742827 | 17446856 |  |
| ROH16 | 8 | 64 | 0.000502094 | 0.9821229 | 1 | 82575142 | 85230072 | 2654930 |  |
| ROH17 | 7 | 76 | 0.57562294 | 0.4480331 | 1 | 85382107 | 88083782 | 2701675 |  |
| ROH18 | 137 | 1214 | 1.307966975 | 0.2527631 | 1 | 88273790 | 107921268 | 19647478 |  |
| ROH19 | 4 | 59 | 1.417177885 | 0.2338685 | 1 | 108223719 | 110283785 | 2060066 |  |
| ROH20 | 261 | 2142 | 0.098228226 | 0.7539663 | 1 | 112688022 | 159135282 | 46447260 | Centromeric |
| ROH21 | 2 | 19 | 0.04860012 | 0.8255173 | 1 | 160676674 | 162299823 | 1623149 |  |
| ROH22 | 0 | 13 | 1.61409983 | 0.2039164 | 1 | 163508344 | 164496611 | 988267 |  |
| ROH23 | 4 | 22 | 0.504386429 | 0.4775791 | 1 | 165769653 | 167373146 | 1603493 |  |
| ROH24 | 160 | 1322 | 0.100355521 | 0.7514034 | 1 | 168585684 | 181325627 | 12739943 |  |
| ROH25 | 266 | 2088 | 0.250429576 | 0.6167728 | 1 | 181500759 | 201424231 | 19923472 |  |
| ROH26 | 0 | 11 | 1.365433188 | 0.2425981 | 1 | 202172769 | 202826538 | 653769 |  |
| ROH27 | 1 | 10 | 0.042156033 | 0.8373226 | 1 | 204978446 | 205315823 | 337377 |  |
| ROH28 | 17 | 123 | 0.180259809 | 0.6711501 | 1 | 205448980 | 208302633 | 2853653 |  |
| ROH29 | 13 | 87 | 0.40104215 | 0.5265515 | 1 | 209412567 | 213565469 | 4152902 |  |
| ROH30 | 12 | 103 | 0.041955425 | 0.8377048 | 1 | 214225713 | 216417771 | 2192058 |  |
| ROH31 | 100 | 805 | 0.000457091 | 0.9829428 | 1 | 217302383 | 230264056 | 12961673 |  |
| ROH32 | 2 | 25 | 0.362080854 | 0.5473528 | 1 | 230436615 | 232091821 | 1655206 |  |
| ROH33 | 1 | 10 | 0.042156033 | 0.8373226 | 1 | 232442369 | 233197806 | 755437 |  |
| ROH34 | 3 | 13 | 0.974071217 | 0.3236669 | 1 | 235095329 | 236190333 | 1095004 |  |
| ROH35 | 4 | 31 | 0.005726696 | 0.9396777 | 1 | 242723279 | 244288525 | 1565246 |  |
| ROH36 | 17 | 136 | 0.001078019 | 0.9738076 | 1 | 246003671 | 249181646 | 3177975 |  |
| ROH37 | 1 | 17 | 0.550761062 | 0.4580069 | 2 | 240785 | 1669549 | 1428764 |  |
| ROH38 | 4 | 14 | 2.303602422 | 0.1290743 | 2 | 8724337 | 9796791 | 1072454 |  |
| ROH39 | 7 | 105 | 2.643785388 | 0.1039556 | 2 | 12631916 | 15999232 | 3367316 |  |
| ROH40 | 7 | 61 | 0.037723852 | 0.8459987 | 2 | 16936078 | 20369811 | 3433733 |  |
| ROH41 | 104 | 922 | 0.955710679 | 0.3282699 | 2 | 20856144 | 30010894 | 9154750 |  |
| ROH42 | 85 | 707 | 0.078765708 | 0.7789771 | 2 | 31283345 | 33595449 | 2312104 |  |
| ROH43 | 7 | 38 | 0.945101822 | 0.3309692 | 2 | 34551566 | 37700786 | 3149220 |  |
| ROH44 | 29 | 324 | 2.979745739 | 0.08431252 | 2 | 38140126 | 43211651 | 5071525 |  |
| ROH45 | 1 | 14 | 0.291423629 | 0.5893097 | 2 | 43245485 | 43412531 | 167046 |  |
| ROH46 | 3 | 29 | 0.089550989 | 0.7647488 | 2 | 43417083 | 45205547 | 1788464 |  |
| ROH47 | 167 | 1299 | 0.241003824 | 0.623482 | 2 | 47552277 | 65005540 | 17453263 |  |
| ROH48 | 2 | 15 | 0.009403432 | 0.9227492 | 2 | 66903422 | 68020044 | 1116622 |  |
| ROH49 | 11 | 143 | 2.416644141 | 0.1200519 | 2 | 69051266 | 70898744 | 1847478 |  |
| ROH50 | 39 | 339 | 0.204622542 | 0.6510153 | 2 | 71297057 | 75384549 | 4087492 |  |
| ROH51 | 6 | 72 | 0.89364575 | 0.3444913 | 2 | 75502853 | 79347745 | 3844892 |  |
| ROH52 | 253 | 2223 | 2.406882926 | 0.1208027 | 2 | 80126697 | 121198408 | 41071711 | Centromeric |
| ROH53 | 47 | 427 | 0.636460813 | 0.4249953 | 2 | 121729360 | 127511804 | 5782444 |  |
| ROH54 | 410 | 3241 | 0.277759081 | 0.5981738 | 2 | 127600704 | 169386870 | 41786166 |  |
| ROH55 | 7 | 47 | 0.207288341 | 0.6489011 | 2 | 171450353 | 173493327 | 2042974 |  |
| ROH56 | 16 | 174 | 1.348923301 | 0.2454664 | 2 | 174627064 | 177235473 | 2608409 |  |
| ROH57 | 192 | 1564 | 0.020644984 | 0.8857503 | 2 | 177338662 | 192366645 | 15027983 |  |
| ROH58 | 250 | 2099 | 0.505816122 | 0.4769557 | 2 | 192980006 | 206443038 | 13463032 |  |
| ROH59 | 5 | 28 | 0.574004631 | 0.448672 | 2 | 206649685 | 208429351 | 1779666 |  |
| ROH60 | 65 | 580 | 0.647236026 | 0.4211026 | 2 | 208555031 | 216313591 | 7758560 |  |
| ROH61 | 21 | 236 | 2.210031057 | 0.1371159 | 2 | 218732851 | 220535982 | 1803131 |  |
| ROH62 | 14 | 106 | 0.050525203 | 0.8221519 | 2 | 223348008 | 227908665 | 4560657 |  |
| ROH63 | 4 | 14 | 2.303602422 | 0.1290743 | 2 | 228808696 | 229883422 | 1074726 |  |
| ROH64 | 1 | 12 | 0.147722896 | 0.700721 | 2 | 230524304 | 231442496 | 918192 |  |
| ROH65 | 3 | 30 | 0.126819045 | 0.7217532 | 2 | 231785313 | 234203597 | 2418284 |  |
| ROH66 | 1 | 11 | 0.088984956 | 0.7654716 | 2 | 237691825 | 238110759 | 418934 |  |
| ROH67 | 2 | 15 | 0.009403432 | 0.9227492 | 2 | 241695096 | 243000278 | 1305182 |  |
| ROH68 | 6 | 35 | 0.545949091 | 0.4599776 | 3 | 11781002 | 13080976 | 1299974 |  |
| ROH69 | 50 | 412 | 0.021263572 | 0.8840632 | 3 | 14846758 | 21486630 | 6639872 |  |
| ROH70 | 7 | 96 | 1.905200859 | 0.1674973 | 3 | 22135039 | 24253228 | 2118189 |  |
| ROH71 | 24 | 140 | 2.218246403 | 0.1363879 | 3 | 25161230 | 27976048 | 2814818 |  |
| ROH72 | 6 | 22 | 3.097149602 | 0.0784295 | 3 | 30592139 | 31845976 | 1253837 |  |
| ROH73 | 407 | 3339 | 0.193559823 | 0.6599702 | 3 | 32455240 | 54794720 | 22339480 |  |
| ROH74 | 34 | 294 | 0.153207807 | 0.6954885 | 3 | 55313400 | 59750226 | 4436826 |  |
| ROH75 | 3 | 13 | 0.974071217 | 0.3236669 | 3 | 61549295 | 62649666 | 1100371 |  |
| ROH76 | 9 | 67 | 0.051730575 | 0.8200788 | 3 | 67102024 | 71012580 | 3910556 |  |
| ROH77 | 476 | 3961 | 0.924247778 | 0.3363621 | 3 | 74206048 | 116366738 | 42160690 | Centromeric |
| ROH78 | 153 | 1246 | 0.014762104 | 0.9032955 | 3 | 116964048 | 133399702 | 16435654 |  |
| ROH79 | 70 | 629 | 0.803676208 | 0.3699965 | 3 | 133696285 | 142927446 | 9231161 |  |
| ROH80 | 15 | 156 | 0.907088247 | 0.3408882 | 3 | 143424822 | 148637771 | 5212949 |  |
| ROH81 | 205 | 1722 | 0.395036287 | 0.5296639 | 3 | 150625280 | 170823923 | 20198643 |  |
| ROH82 | 1 | 34 | 2.392333204 | 0.1219315 | 3 | 172786148 | 175027803 | 2241655 |  |
| ROH83 | 3 | 26 | 0.013875205 | 0.9062316 | 3 | 175109003 | 176672414 | 1563411 |  |
| ROH84 | 58 | 467 | 0.000203615 | 0.9886151 | 3 | 177113056 | 185893790 | 8780734 |  |
| ROH85 | 2 | 13 | 0.081341636 | 0.7754874 | 3 | 190961156 | 192055150 | 1093994 |  |
| ROH86 | 26 | 183 | 0.435752572 | 0.5091797 | 3 | 194612501 | 3644957 |  | Removed from analysis |
| ROH87 | 72 | 555 | 0.143272644 | 0.705049 | 4 | 8557865 | 11057066 | 2499201 |  |
| ROH88 | 6 | 14 | 7.35422835 | 0.006690522 | 4 | 11631446 | 12875351 | 1243905 |  |
| ROH89 | 2 | 31 | 0.833551457 | 0.3612476 | 4 | 14320086 | 15964349 | 1644263 |  |
| ROH90 | 25 | 206 | 0.01031407 | 0.9191074 | 4 | 17420951 | 20536493 | 3115542 |  |
| ROH91 | 7 | 43 | 0.452692887 | 0.5010589 | 4 | 21382332 | 23815878 | 2433546 |  |
| ROH92 | 3 | 11 | 1.545846721 | 0.2137496 | 4 | 26166250 | 27216751 | 1050501 |  |
| ROH93 | 255 | 1984 | 0.412002489 | 0.5209549 | 4 | 27235976 | 37319418 | 10083442 |  |
| ROH94 | 5 | 50 | 0.21195324 | 0.6452408 | 4 | 38465656 | 40345790 | 1880134 |  |
| ROH95 | 78 | 556 | 1.139576302 | 0.2857419 | 4 | 41225831 | 54866124 | 13640293 | Centromeric |
| ROH96 | 0 | 26 | 3.233488457 | 0.07214685 | 4 | 55888635 | 57096491 | 1207856 |  |
| ROH97 | 294 | 2356 | 0.017407213 | 0.8950346 | 4 | 58353664 | 83946075 | 25592411 |  |
| ROH98 | 173 | 1548 | 2.133561924 | 0.1441055 | 4 | 84778595 | 111143870 | 26365275 |  |
| ROH99 | 13 | 95 | 0.11343172 | 0.7362705 | 4 | 111278172 | 113960961 | 2682789 |  |
| ROH100 | 165 | 1426 | 0.877162967 | 0.3489799 | 4 | 114122971 | 139153535 | 25030564 |  |
| ROH101 | 150 | 1220 | 0.010755245 | 0.9174015 | 4 | 139557368 | 153879878 | 14322510 |  |
| ROH102 | 2 | 16 | 0.00012467 | 0.9910913 | 4 | 154766244 | 156038542 | 1272298 |  |
| ROH103 | 45 | 448 | 1.935472815 | 0.1641609 | 4 | 156677592 | 169596649 | 12919057 |  |
| ROH104 | 69 | 670 | 2.376228127 | 0.1231947 | 4 | 169662419 | 174476098 | 4813679 |  |
| ROH105 | 1 | 22 | 1.049259445 | 0.3056777 | 4 | 175094250 | 176591669 | 1497419 |  |
| ROH106 | 0 | 17 | 2.111808745 | 0.1461665 | 4 | 178209089 | 179398739 | 1189650 |  |
| ROH107 | 1 | 22 | 1.049259445 | 0.3056777 | 4 | 190872778 | 1140672 |  | Removed from analysis |
| ROH108 | 78 | 676 | 0.397046118 | 0.5286187 | 5 | 11399734 | 13761068 | 2361334 |  |
| ROH109 | 11 | 140 | 2.198811582 | 0.1381172 | 5 | 14460515 | 16480810 | 2020295 |  |
| ROH110 | 84 | 683 | 0.005129267 | 0.9429052 | 5 | 17391377 | 31144547 | 13753170 |  |
| ROH111 | 22 | 212 | 0.650430607 | 0.4199588 | 5 | 32058096 | 38129168 | 6071072 |  |
| ROH112 | 173 | 1483 | 0.721557627 | 0.3956335 | 5 | 38378483 | 53721698 | 15343215 | Centromeric |
| ROH113 | 21 | 146 | 0.415541072 | 0.5191704 | 5 | 53921870 | 55699243 | 1777373 |  |
| ROH114 | 126 | 1013 | 0.001526545 | 0.9688338 | 5 | 55872943 | 67098647 | 11225704 |  |
| ROH115 | 1 | 19 | 0.742681589 | 0.3888032 | 5 | 67644040 | 68813626 | 1169586 |  |
| ROH116 | 6 | 45 | 0.028331554 | 0.8663317 | 5 | 71184918 | 72772062 | 1587144 |  |
| ROH117 | 35 | 333 | 0.905960733 | 0.3411884 | 5 | 73937506 | 75714250 | 1776744 |  |
| ROH118 | 3 | 22 | 0.024108928 | 0.8766081 | 5 | 77142481 | 79050825 | 1908344 |  |
| ROH119 | 296 | 2515 | 1.101366206 | 0.2939665 | 5 | 79247901 | 115091714 | 35843813 |  |
| ROH120 | 31 | 263 | 0.073044206 | 0.7869549 | 5 | 116006026 | 123620112 | 7614086 |  |
| ROH121 | 190 | 1616 | 0.615011688 | 0.432907 | 5 | 125874334 | 132852187 | 6977853 |  |
| ROH122 | 6 | 75 | 1.093686355 | 0.2956559 | 5 | 133021851 | 134612693 | 1590842 |  |
| ROH123 | 1 | 9 | 0.010799457 | 0.9172325 | 5 | 134618432 | 134778470 | 160038 |  |
| ROH124 | 92 | 715 | 0.126296893 | 0.7223029 | 5 | 134957413 | 141394893 | 6437480 |  |
| ROH125 | 52 | 379 | 0.498609508 | 0.4801117 | 5 | 141988810 | 146594950 | 4606140 |  |
| ROH126 | 2 | 22 | 0.178238986 | 0.672891 | 5 | 146951712 | 148309976 | 1358264 |  |
| ROH127 | 60 | 464 | 0.101615442 | 0.7498996 | 5 | 150722675 | 165172627 | 14449952 |  |
| ROH128 | 0 | 14 | 1.738480088 | 0.1873315 | 5 | 165235055 | 166302658 | 1067603 |  |
| ROH129 | 4 | 59 | 1.417177885 | 0.2338685 | 5 | 175131097 | 177751488 | 2620391 |  |
| ROH130 | 2 | 17 | 0.004900039 | 0.9441934 | 6 | 14988767 | 16098290 | 1109523 |  |
| ROH131 | 18 | 97 | 2.541939554 | 0.1108585 | 6 | 16835621 | 19667419 | 2831798 |  |
| ROH132 | 0 | 18 | 2.236314298 | 0.134802 | 6 | 20209344 | 21357289 | 1147945 |  |
| ROH133 | 1 | 12 | 0.147722896 | 0.700721 | 6 | 22220860 | 23315977 | 1095117 |  |
| ROH134 | 294 | 2239 | 1.264969094 | 0.2607126 | 6 | 23909796 | 36731875 | 12822079 |  |
| ROH135 | 10 | 66 | 0.354492594 | 0.5515809 | 6 | 37569632 | 39796910 | 2227278 |  |
| ROH136 | 9 | 43 | 2.103021732 | 0.1470084 | 6 | 42207784 | 43716391 | 1508607 |  |
| ROH137 | 10 | 67 | 0.303857877 | 0.5814739 | 6 | 44246766 | 45885698 | 1638932 |  |
| ROH138 | 65 | 470 | 0.73421118 | 0.3915216 | 6 | 45991569 | 53130285 | 7138716 |  |
| ROH139 | 359 | 3056 | 1.624764848 | 0.2024287 | 6 | 53307694 | 91106000 | 37798306 | Centromeric |
| ROH140 | 113 | 898 | 0.025951637 | 0.8720185 | 6 | 91500635 | 106123956 | 14623321 |  |
| ROH141 | 158 | 1223 | 0.289345076 | 0.5906405 | 6 | 107694996 | 124180018 | 16485022 |  |
| ROH142 | 57 | 437 | 0.139442412 | 0.708836 | 6 | 124474595 | 134428278 | 9953683 |  |
| ROH143 | 16 | 119 | 0.094814798 | 0.7581431 | 6 | 134658911 | 138809476 | 4150565 |  |
| ROH144 | 100 | 751 | 0.50636667 | 0.4767159 | 6 | 139411399 | 148449589 | 9038190 |  |
| ROH145 | 4 | 47 | 0.530853787 | 0.4662481 | 6 | 149112190 | 150670899 | 1558709 |  |
| ROH146 | 2 | 20 | 0.084428886 | 0.7713829 | 6 | 158163207 | 159516770 | 1353563 |  |
| ROH147 | 1 | 9 | 0.010799457 | 0.9172325 | 6 | 160067287 | 160241959 | 174672 |  |
| ROH148 | 5 | 25 | 0.975482263 | 0.3233167 | 6 | 160251641 | 161698258 | 1446617 |  |
| ROH149 | 2 | 12 | 0.151613498 | 0.6969982 | 6 | 162646892 | 163613216 | 966324 |  |
| ROH150 | 2 | 10 | 0.389309338 | 0.5326627 | 6 | 164752315 | 165790374 | 1038059 |  |
| ROH151 | 0 | 23 | 2.859312609 | 0.09084631 | 6 | 169542869 | 170890384 | 1347515 |  |
| ROH152 | 6 | 31 | 1.019427591 | 0.3126548 | 7 | 420303 | 2608271 | 2187968 |  |
| ROH153 | 4 | 26 | 0.162990958 | 0.6864176 | 7 | 3063921 | 4413590 | 1349669 |  |
| ROH154 | 4 | 31 | 0.005726696 | 0.9396777 | 7 | 5516940 | 7171376 | 1654436 |  |
| ROH155 | 5 | 42 | 0.007346194 | 0.931697 | 7 | 9648613 | 11316581 | 1667968 |  |
| ROH156 | 3 | 16 | 0.439904095 | 0.5071687 | 7 | 11806005 | 12953229 | 1147224 |  |
| ROH157 | 5 | 21 | 1.788370447 | 0.1811248 | 7 | 15113866 | 16785610 | 1671744 |  |
| ROH158 | 9 | 45 | 1.761197634 | 0.1844752 | 7 | 17554444 | 20193979 | 2639535 |  |
| ROH159 | 18 | 155 | 0.070206389 | 0.7910365 | 7 | 22158627 | 28367949 | 6209322 |  |
| ROH160 | 4 | 38 | 0.097458001 | 0.7549017 | 7 | 29365366 | 31131353 | 1765987 |  |
| ROH161 | 23 | 184 | 0.001468654 | 0.9694301 | 7 | 31719444 | 36396358 | 4676914 |  |
| ROH162 | 10 | 101 | 0.46799622 | 0.4939104 | 7 | 38357048 | 41360478 | 3003430 |  |
| ROH163 | 9 | 64 | 0.126892233 | 0.7216763 | 7 | 42233283 | 46672692 | 4439409 |  |
| ROH164 | 197 | 1514 | 0.522383955 | 0.4698265 | 7 | 47806077 | 70434205 | 22628128 | Centromeric |
| ROH165 | 24 | 164 | 0.592183149 | 0.4415761 | 7 | 70860252 | 77612346 | 6752094 |  |
| ROH166 | 5 | 46 | 0.078477674 | 0.7793711 | 7 | 78407722 | 80114607 | 1706885 |  |
| ROH167 | 249 | 1994 | 0.016699955 | 0.897177 | 7 | 82183868 | 105356897 | 23173029 |  |
| ROH168 | 354 | 3071 | 2.942006434 | 0.08630347 | 7 | 106312107 | 130537598 | 24225491 |  |
| ROH169 | 29 | 190 | 1.124192321 | 0.2890175 | 7 | 131978162 | 135609081 | 3630919 |  |
| ROH170 | 6 | 29 | 1.339272702 | 0.2471623 | 7 | 135870953 | 137682885 | 1811932 |  |
| ROH171 | 4 | 64 | 1.85125854 | 0.1736371 | 7 | 140104719 | 141819278 | 1714559 |  |
| ROH172 | 24 | 176 | 0.197220415 | 0.6569739 | 7 | 141850772 | 146942272 | 5091500 |  |
| ROH173 | 3 | 14 | 0.760488862 | 0.3831756 | 7 | 157789039 | 159070969 | 1281930 |  |
| ROH174 | 38 | 261 | 0.893872651 | 0.34443 | 8 | 6801308 | 12973575 | 6172267 |  |
| ROH175 | 5 | 40 | 0.000312739 | 0.9858906 | 8 | 13736874 | 16637512 | 2900638 |  |
| ROH176 | 2 | 8 | 0.821247016 | 0.3648158 | 8 | 17626446 | 18618764 | 992318 |  |
| ROH177 | 1 | 16 | 0.459843393 | 0.4976972 | 8 | 19827848 | 21176295 | 1348447 |  |
| ROH178 | 13 | 77 | 1.082325435 | 0.2981779 | 8 | 23547067 | 27827391 | 4280324 |  |
| ROH179 | 0 | 12 | 1.489750868 | 0.2222551 | 8 | 28308594 | 29256738 | 948144 |  |
| ROH180 | 127 | 976 | 0.307303207 | 0.5793398 | 8 | 29746412 | 40482871 | 10736459 |  |
| ROH181 | 531 | 4218 | 0.279102034 | 0.5972904 | 8 | 40881755 | 70132590 | 29250835 | Centromeric |
| ROH182 | 17 | 59 | 10.05547881 | 0.001518955 | 8 | 70564701 | 72329553 | 1764852 |  |
| ROH183 | 269 | 2090 | 0.478830664 | 0.4889525 | 8 | 73240030 | 98902730 | 25662700 |  |
| ROH184 | 20 | 159 | 0.003892265 | 0.9502538 | 8 | 99044528 | 101662721 | 2618193 |  |
| ROH185 | 1 | 14 | 0.291423629 | 0.5893097 | 8 | 102495233 | 103526633 | 1031400 |  |
| ROH186 | 170 | 1370 | 0.00020228 | 0.9886525 | 8 | 103794864 | 122908503 | 19113639 |  |
| ROH187 | 6 | 48 | 0.000375714 | 0.9845353 | 8 | 129169758 | 131756936 | 2587178 |  |
| ROH188 | 6 | 49 | 0.000808349 | 0.977318 | 8 | 136212232 | 139028105 | 2815873 |  |
| ROH189 | 9 | 97 | 0.705345976 | 0.4009932 | 8 | 143193865 | 146278197 | 3084332 |  |
| ROH190 | 6 | 57 | 0.146575698 | 0.7018295 | 9 | 4766022 | 7129452 | 2363430 |  |
| ROH191 | 28 | 221 | 0.012245988 | 0.9118847 | 9 | 10583223 | 13689066 | 3105843 |  |
| ROH192 | 3 | 37 | 0.510172005 | 0.4750644 | 9 | 15083950 | 18140836 | 3056886 |  |
| ROH193 | 9 | 52 | 0.869408911 | 0.351119 | 9 | 20289153 | 23354494 | 3065341 |  |
| ROH194 | 4 | 45 | 0.412573869 | 0.520666 | 9 | 23427062 | 25519176 | 2092114 |  |
| ROH195 | 8 | 59 | 0.05721552 | 0.8109523 | 9 | 25598674 | 27358115 | 1759441 |  |
| ROH196 | 43 | 279 | 1.849089845 | 0.1738893 | 9 | 27962499 | 32870789 | 4908290 |  |
| ROH197 | 34 | 327 | 0.99953232 | 0.3174237 | 9 | 32884738 | 38130114 | 5245376 |  |
| ROH198 | 11 | 75 | 0.275053711 | 0.5999617 | 9 | 71387764 | 74278480 | 2890716 |  |
| ROH199 | 23 | 184 | 0.001468654 | 0.9694301 | 9 | 74292618 | 77624761 | 3332143 |  |
| ROH200 | 1 | 29 | 1.817399554 | 0.1776231 | 9 | 79691650 | 81254538 | 1562888 |  |
| ROH201 | 6 | 54 | 0.065206796 | 0.798448 | 9 | 82299047 | 84202956 | 1903909 |  |
| ROH202 | 6 | 58 | 0.179827731 | 0.6715213 | 9 | 84256984 | 85813844 | 1556860 |  |
| ROH203 | 6 | 59 | 0.215911002 | 0.6421733 | 9 | 86108400 | 89230779 | 3122379 |  |
| ROH204 | 3 | 30 | 0.126819045 | 0.7217532 | 9 | 90362580 | 92195490 | 1832910 |  |
| ROH205 | 84 | 679 | 0.000330076 | 0.9855048 | 9 | 93837417 | 100933383 | 7095966 |  |
| ROH206 | 12 | 148 | 2.072079194 | 0.1500169 | 9 | 101633298 | 104160157 | 2526859 |  |
| ROH207 | 18 | 76 | 6.392049964 | 0.01146326 | 9 | 104341634 | 107664301 | 3322667 |  |
| ROH208 | 18 | 123 | 0.441467994 | 0.5064148 | 9 | 107684276 | 109382361 | 1698085 |  |
| ROH209 | 5 | 70 | 1.468204509 | 0.2256292 | 9 | 110943815 | 115785680 | 4841865 |  |
| ROH210 | 10 | 66 | 0.354492594 | 0.5515809 | 9 | 117453668 | 121370958 | 3917290 |  |
| ROH211 | 45 | 312 | 0.946416235 | 0.3306332 | 9 | 122656864 | 124747323 | 2090459 |  |
| ROH212 | 38 | 331 | 0.21052061 | 0.6463597 | 9 | 124878277 | 129121098 | 4242821 |  |
| ROH213 | 42 | 311 | 0.284437256 | 0.5938075 | 9 | 129270986 | 132162470 | 2891484 |  |
| ROH214 | 5 | 28 | 0.574004631 | 0.448672 | 9 | 141098428 | 1340620 |  | Removed from analysis |
| ROH215 | 7 | 38 | 0.945101822 | 0.3309692 | 10 | 8768500 | 10628982 | 1860482 |  |
| ROH216 | 5 | 15 | 3.98855417 | 0.04581036 | 10 | 15269846 | 16556710 | 1286864 |  |
| ROH217 | 7 | 17 | 8.070864397 | 0.004498265 | 10 | 17248287 | 18664854 | 1416567 |  |
| ROH218 | 46 | 395 | 0.17010108 | 0.680022 | 10 | 18742477 | 24502070 | 5759593 |  |
| ROH219 | 18 | 120 | 0.580929273 | 0.4459483 | 10 | 24698015 | 29005257 | 4307242 |  |
| ROH220 | 33 | 267 | 0.000262883 | 0.9870639 | 10 | 30791941 | 33855696 | 3063755 |  |
| ROH221 | 113 | 881 | 0.133373032 | 0.7149601 | 10 | 34178427 | 53220004 | 19041577 | Centromeric |
| ROH222 | 235 | 1663 | 4.647245621 | 0.03110342 | 10 | 54564689 | 71149319 | 16584630 |  |
| ROH223 | 231 | 1784 | 0.520868436 | 0.4704715 | 10 | 73434602 | 79173697 | 5739095 |  |
| ROH224 | 4 | 8 | 6.093228986 | 0.01357009 | 10 | 79214413 | 80017045 | 802632 |  |
| ROH225 | 5 | 79 | 2.229556772 | 0.1353927 | 10 | 81173300 | 82547572 | 1374272 |  |
| ROH226 | 34 | 353 | 2.086058688 | 0.1486491 | 10 | 82671690 | 90904111 | 8232421 |  |
| ROH227 | 33 | 276 | 0.03993996 | 0.841598 | 10 | 91643014 | 95503759 | 3860745 |  |
| ROH228 | 215 | 1602 | 1.550697204 | 0.2130325 | 10 | 95515515 | 114258451 | 18742936 |  |
| ROH229 | 13 | 120 | 0.216877731 | 0.6414293 | 10 | 116048058 | 119167736 | 3119678 |  |
| ROH230 | 1 | 24 | 1.262663245 | 0.2611476 | 10 | 119626837 | 121901799 | 2274962 |  |
| ROH231 | 1 | 21 | 0.945021438 | 0.3309898 | 11 | 3397231 | 5016269 | 1619038 |  |
| ROH232 | 19 | 114 | 1.462223869 | 0.2265766 | 11 | 8239818 | 10849613 | 2609795 |  |
| ROH233 | 26 | 182 | 0.471138533 | 0.4924639 | 11 | 12868119 | 17550577 | 4682458 |  |
| ROH234 | 2 | 17 | 0.004900039 | 0.9441934 | 11 | 17580998 | 18744695 | 1163697 |  |
| ROH235 | 2 | 28 | 0.583949603 | 0.4447684 | 11 | 22099583 | 24610850 | 2511267 |  |
| ROH236 | 57 | 483 | 0.132199458 | 0.7161624 | 11 | 24848824 | 33718074 | 8869250 |  |
| ROH237 | 120 | 937 | 0.130490161 | 0.7179244 | 11 | 36873565 | 44033733 | 7160168 |  |
| ROH238 | 374 | 3166 | 1.397407718 | 0.2371581 | 11 | 44966113 | 68995044 | 24028931 | Centromeric |
| ROH239 | 55 | 478 | 0.293317422 | 0.5881024 | 11 | 70496641 | 76350119 | 5853478 |  |
| ROH240 | 12 | 107 | 0.1098824 | 0.7402781 | 11 | 76438258 | 78909461 | 2471203 |  |
| ROH241 | 132 | 1056 | 0.009649621 | 0.9217477 | 11 | 80818238 | 94854662 | 14036424 |  |
| ROH242 | 79 | 632 | 0.005395187 | 0.9414465 | 11 | 95712558 | 110826882 | 15114324 |  |
| ROH243 | 5 | 94 | 3.651261125 | 0.05602677 | 11 | 111111729 | 113476298 | 2364569 |  |
| ROH244 | 4 | 33 | 0.001799616 | 0.9661624 | 11 | 114271207 | 115976508 | 1705301 |  |
| ROH245 | 1 | 17 | 0.550761062 | 0.4580069 | 11 | 116479752 | 117709625 | 1229873 |  |
| ROH246 | 7 | 34 | 1.534464953 | 0.2154436 | 11 | 117774829 | 119635453 | 1860624 |  |
| ROH247 | 6 | 17 | 5.33032237 | 0.02095751 | 11 | 123470475 | 124699080 | 1228605 |  |
| ROH248 | 2 | 18 | 0.021626113 | 0.8830861 | 11 | 124723756 | 126246076 | 1522320 |  |
| ROH249 | 1 | 11 | 0.088984956 | 0.7654716 | 11 | 127184582 | 128119255 | 934673 |  |
| ROH250 | 14 | 66 | 3.448848246 | 0.06329586 | 12 | 8692843 | 11934852 | 3242009 |  |
| ROH251 | 14 | 166 | 1.983621425 | 0.1590094 | 12 | 14056953 | 20081721 | 6024768 |  |
| ROH252 | 11 | 71 | 0.480647872 | 0.488129 | 12 | 20533594 | 24274071 | 3740477 |  |
| ROH253 | 5 | 42 | 0.007346194 | 0.931697 | 12 | 25479948 | 27882080 | 2402132 |  |
| ROH254 | 5 | 44 | 0.034172412 | 0.8533406 | 12 | 27911100 | 30658643 | 2747543 |  |
| ROH255 | 225 | 1762 | 0.23458014 | 0.6281483 | 12 | 31066094 | 52211617 | 21145523 | Centromeric |
| ROH256 | 112 | 929 | 0.088894662 | 0.7655871 | 12 | 52589512 | 67321354 | 14731842 |  |
| ROH257 | 77 | 632 | 0.02249345 | 0.8807818 | 12 | 69363979 | 76109814 | 6745835 |  |
| ROH258 | 210 | 1803 | 0.97722205 | 0.3228856 | 12 | 76417290 | 93818928 | 17401638 |  |
| ROH259 | 1 | 12 | 0.147722896 | 0.700721 | 12 | 96029362 | 96440613 | 411251 |  |
| ROH260 | 2 | 28 | 0.583949603 | 0.4447684 | 12 | 96451981 | 97690255 | 1238274 |  |
| ROH261 | 16 | 160 | 0.688792217 | 0.4065755 | 12 | 98190477 | 104143173 | 5952696 |  |
| ROH262 | 6 | 71 | 0.830045427 | 0.3622594 | 12 | 106481727 | 108502877 | 2021150 |  |
| ROH263 | 233 | 1775 | 0.90423919 | 0.3416476 | 12 | 108874961 | 113966601 | 5091640 |  |
| ROH264 | 78 | 681 | 0.483630939 | 0.4867822 | 12 | 118509138 | 124858380 | 6349242 |  |
| ROH265 | 0 | 17 | 2.111808745 | 0.1461665 | 12 | 132026695 | 133654579 | 1627884 |  |
| ROH266 | 30 | 275 | 0.459781208 | 0.4977262 | 12 | 133814152 | 20910048 |  | Removed from analysis |
| ROH267 | 8 | 63 | 0.004164742 | 0.9485444 | 13 | 32802838 | 36476447 | 3673609 |  |
| ROH268 | 5 | 48 | 0.13829752 | 0.7099794 | 13 | 37460648 | 40092849 | 2632201 |  |
| ROH269 | 14 | 65 | 3.642432127 | 0.0563246 | 13 | 40913357 | 43405061 | 2491704 |  |
| ROH270 | 3 | 34 | 0.323153989 | 0.5697183 | 13 | 44663145 | 46494486 | 1831341 |  |
| ROH271 | 219 | 1699 | 0.390419442 | 0.532079 | 13 | 47469940 | 74166665 | 26696725 |  |
| ROH272 | 116 | 936 | 5.7065E-06 | 0.998094 | 13 | 75894015 | 92212151 | 16318136 |  |
| ROH273 | 2 | 38 | 1.489113621 | 0.222354 | 13 | 92554839 | 94827624 | 2272785 |  |
| ROH274 | 72 | 606 | 0.126527793 | 0.7220597 | 13 | 95847161 | 98433916 | 2586755 |  |
| ROH275 | 6 | 68 | 0.649339979 | 0.4203488 | 13 | 99199942 | 101604450 | 2404508 |  |
| ROH276 | 1 | 25 | 1.371457235 | 0.2415617 | 13 | 102281844 | 104075992 | 1794148 |  |
| ROH277 | 2 | 19 | 0.04860012 | 0.8255173 | 13 | 113681076 | 115090019 | 1408943 |  |
| ROH278 | 1 | 15 | 0.373120049 | 0.5413085 | 14 | 24308557 | 25630912 | 1322355 |  |
| ROH279 | 10 | 71 | 0.14463207 | 0.7037189 | 14 | 27517393 | 32837088 | 5319695 |  |
| ROH280 | 15 | 153 | 0.771461868 | 0.3797652 | 14 | 34771929 | 37151655 | 2379726 |  |
| ROH281 | 199 | 1677 | 0.44351859 | 0.5054291 | 14 | 37190198 | 51395083 | 14204885 |  |
| ROH282 | 5 | 42 | 0.007346194 | 0.931697 | 14 | 52324354 | 54184670 | 1860316 |  |
| ROH283 | 8 | 77 | 0.228941569 | 0.6323098 | 14 | 55080682 | 56511463 | 1430781 |  |
| ROH284 | 295 | 2543 | 1.807272242 | 0.1788358 | 14 | 57384944 | 72903279 | 15518335 |  |
| ROH285 | 10 | 139 | 2.884369116 | 0.08944303 | 14 | 72939495 | 75884983 | 2945488 |  |
| ROH286 | 30 | 292 | 1.00234097 | 0.3167447 | 14 | 77481725 | 89550640 | 12068915 |  |
| ROH287 | 2 | 15 | 0.009403432 | 0.9227492 | 14 | 91306972 | 92729907 | 1422935 |  |
| ROH288 | 0 | 21 | 2.610019129 | 0.1061905 | 14 | 93070367 | 94317328 | 1246961 |  |
| ROH289 | 17 | 157 | 0.287197296 | 0.5920222 | 14 | 101757475 | 104764853 | 3007378 |  |
| ROH290 | 7 | 58 | 0.004498704 | 0.9465241 | 14 | 105037425 | 107274052 | 2236627 |  |
| ROH291 | 2 | 25 | 0.362080854 | 0.5473528 | 15 | 23999992 | 25115982 | 1115990 |  |
| ROH292 | 44 | 283 | 2.046857954 | 0.1525207 | 15 | 27672555 | 33185581 | 5513026 |  |
| ROH293 | 265 | 2187 | 0.17678652 | 0.6741495 | 15 | 39374881 | 53682712 | 14307831 |  |
| ROH294 | 43 | 435 | 2.141886109 | 0.1433256 | 15 | 54133298 | 57832969 | 3699671 |  |
| ROH295 | 5 | 31 | 0.301483789 | 0.5829537 | 15 | 58723675 | 60001060 | 1277385 |  |
| ROH296 | 3 | 15 | 0.584405124 | 0.4445908 | 15 | 61538915 | 62720363 | 1181448 |  |
| ROH297 | 16 | 207 | 3.472666097 | 0.06239068 | 15 | 63019111 | 66891941 | 3872830 |  |
| ROH298 | 43 | 215 | 8.637456643 | 0.003293209 | 15 | 67138238 | 68752965 | 1614727 |  |
| ROH299 | 12 | 57 | 2.871534636 | 0.09015886 | 15 | 68881524 | 70090174 | 1208650 |  |
| ROH300 | 237 | 1703 | 3.687732302 | 0.05481409 | 15 | 70546312 | 78949127 | 8402815 |  |
| ROH301 | 2 | 14 | 0.03536561 | 0.8508316 | 15 | 79487582 | 80694674 | 1207092 |  |
| ROH302 | 60 | 474 | 0.025041238 | 0.8742643 | 15 | 81614805 | 87560258 | 5945453 |  |
| ROH303 | 6 | 31 | 1.019427591 | 0.3126548 | 16 | 1045384 | 4422607 | 3377223 |  |
| ROH304 | 4 | 51 | 0.796263738 | 0.3722128 | 16 | 13938976 | 17279498 | 3340522 |  |
| ROH305 | 16 | 115 | 0.190598374 | 0.6624191 | 16 | 19234492 | 23203830 | 3969338 |  |
| ROH306 | 22 | 220 | 0.955187225 | 0.3284024 | 16 | 27890532 | 32579387 | 4688855 |  |
| ROH307 | 171 | 1415 | 0.123066866 | 0.7257317 | 16 | 35173765 | 49547497 | 14373732 | Centromeric |
| ROH308 | 2 | 23 | 0.234455641 | 0.6282395 | 16 | 49764948 | 51084296 | 1319348 |  |
| ROH309 | 2 | 21 | 0.12796153 | 0.720555 | 16 | 55713786 | 57023938 | 1310152 |  |
| ROH310 | 330 | 2927 | 4.517274488 | 0.03355424 | 16 | 58299711 | 73213224 | 14913513 |  |
| ROH311 | 10 | 67 | 0.303857877 | 0.5814739 | 16 | 73889464 | 77680645 | 3791181 |  |
| ROH312 | 1 | 11 | 0.088984956 | 0.7654716 | 16 | 79699967 | 80659931 | 959964 |  |
| ROH313 | 12 | 52 | 3.919967491 | 0.0477158 | 16 | 88616697 | 90130136 | 1513439 |  |
| ROH314 | 1 | 34 | 2.392333204 | 0.1219315 | 17 | 4439703 | 5713654 | 1273951 |  |
| ROH315 | 46 | 381 | 0.030119023 | 0.8622204 | 17 | 15016491 | 21431819 | 6415328 |  |
| ROH316 | 99 | 832 | 0.167673079 | 0.6821882 | 17 | 22242355 | 31318503 | 9076148 | Centromeric |
| ROH317 | 2 | 24 | 0.295929824 | 0.5864454 | 17 | 32989538 | 34949598 | 1960060 |  |
| ROH318 | 173 | 1388 | 0.005743467 | 0.9395896 | 17 | 36598626 | 47586212 | 10987586 |  |
| ROH319 | 33 | 253 | 0.078541961 | 0.7792831 | 17 | 48990275 | 52976066 | 3985791 |  |
| ROH320 | 2 | 28 | 0.583949603 | 0.4447684 | 17 | 53599731 | 54786262 | 1186531 |  |
| ROH321 | 250 | 2181 | 1.979171932 | 0.1594777 | 17 | 55864209 | 64702429 | 8838220 |  |
| ROH322 | 3 | 11 | 1.545846721 | 0.2137496 | 17 | 68264545 | 69323893 | 1059348 |  |
| ROH323 | 5 | 54 | 0.395195642 | 0.5295809 | 17 | 72768970 | 74794457 | 2025487 |  |
| ROH324 | 2 | 10 | 0.389309338 | 0.5326627 | 18 | 1017323 | 2032309 | 1014986 |  |
| ROH325 | 2 | 20 | 0.084428886 | 0.7713829 | 18 | 12021518 | 13293265 | 1271747 |  |
| ROH326 | 3 | 21 | 0.053101885 | 0.8177511 | 18 | 13516214 | 14902902 | 1386688 |  |
| ROH327 | 92 | 684 | 0.599152209 | 0.4389017 | 18 | 15102421 | 21912975 | 6810554 | Centromeric |
| ROH328 | 7 | 29 | 2.610193414 | 0.1061788 | 18 | 22908330 | 24091430 | 1183100 |  |
| ROH329 | 40 | 267 | 1.30238735 | 0.2537776 | 18 | 25251856 | 28787062 | 3535206 |  |
| ROH330 | 61 | 555 | 0.862951243 | 0.3529141 | 18 | 30028088 | 43899499 | 13871411 |  |
| ROH331 | 6 | 32 | 0.881918041 | 0.3476769 | 18 | 43948347 | 45544934 | 1596587 |  |
| ROH332 | 53 | 451 | 0.144247974 | 0.7040939 | 18 | 46303538 | 55238725 | 8935187 |  |
| ROH333 | 7 | 60 | 0.023244869 | 0.878822 | 18 | 57622287 | 59100766 | 1478479 |  |
| ROH334 | 3 | 29 | 0.089550989 | 0.7647488 | 18 | 59391297 | 60776578 | 1385281 |  |
| ROH335 | 15 | 109 | 0.146424162 | 0.7019762 | 18 | 61188306 | 65109829 | 3921523 |  |
| ROH336 | 1 | 13 | 0.21582462 | 0.6422399 | 18 | 66156903 | 67227276 | 1070373 |  |
| ROH337 | 4 | 29 | 0.040303437 | 0.8408885 | 18 | 67372945 | 69297624 | 1924679 |  |
| ROH338 | 22 | 169 | 0.047798494 | 0.8269393 | 19 | 9227240 | 13355633 | 4128393 |  |
| ROH339 | 51 | 352 | 1.144200525 | 0.2847665 | 19 | 18440960 | 30581807 | 12140847 | Centromeric |
| ROH340 | 10 | 45 | 2.887884857 | 0.08924802 | 19 | 30896106 | 33718053 | 2821947 |  |
| ROH341 | 196 | 1650 | 0.414153793 | 0.5198687 | 19 | 35978299 | 45298069 | 9319770 |  |
| ROH342 | 0 | 13 | 1.61409983 | 0.2039164 | 19 | 57630475 | 58694015 | 1063540 |  |
| ROH343 | 3 | 19 | 0.152872514 | 0.6958053 | 20 | 11138229 | 12286659 | 1148430 |  |
| ROH344 | 15 | 72 | 3.459901296 | 0.06287407 | 20 | 13243610 | 15164186 | 1920576 |  |
| ROH345 | 4 | 26 | 0.162990958 | 0.6864176 | 20 | 17830181 | 19088878 | 1258697 |  |
| ROH346 | 392 | 2851 | 6.25193649 | 0.01240576 | 20 | 20341191 | 40741888 | 20400697 | Centromeric |
| ROH347 | 6 | 39 | 0.244949713 | 0.6206538 | 20 | 43332582 | 45121949 | 1789367 |  |
| ROH348 | 4 | 48 | 0.593805323 | 0.4409514 | 20 | 46918537 | 48655016 | 1736479 |  |
| ROH349 | 6 | 55 | 0.089116074 | 0.7653039 | 20 | 62910548 | 15957477 |  | Removed from analysis |
| ROH350 | 3 | 13 | 0.974071217 | 0.3236669 | 21 | 20655418 | 21813380 | 1157962 |  |
| ROH351 | 14 | 94 | 0.417427457 | 0.5182235 | 21 | 22065557 | 27824285 | 5758728 |  |
| ROH352 | 77 | 758 | 3.104904293 | 0.07805682 | 21 | 28608163 | 32721654 | 4113491 |  |
| ROH353 | 1 | 16 | 0.459843393 | 0.4976972 | 21 | 34620801 | 35914856 | 1294055 |  |
| ROH354 | 0 | 15 | 1.862891651 | 0.1722914 | 21 | 36816352 | 37620240 | 803888 |  |
| ROH355 | 2 | 15 | 0.009403432 | 0.9227492 | 21 | 44753262 | 45868239 | 1114977 |  |
| ROH356 | 8 | 44 | 1.012089093 | 0.3144029 | 22 | 18545634 | 23255525 | 4709891 |  |
| ROH357 | 1 | 30 | 1.931071018 | 0.1646413 | 22 | 23960187 | 25397740 | 1437553 |  |
| ROH358 | 143 | 1108 | 0.244552796 | 0.620937 | 22 | 28141378 | 32859702 | 4718324 |  |
| ROH359 | 3 | 32 | 0.216338797 | 0.6418438 | 22 | 34061053 | 35890089 | 1829036 |  |
| ROH360 | 6 | 20 | 3.858858274 | 0.04948399 | 22 | 37761994 | 39286399 | 1524405 |  |
| ROH361 | 85 | 689 | 0.001918054 | 0.9650673 | 22 | 39685832 | 43538133 | 3852301 |  |
| ROH362 | 3 | 11 | 1.545846721 | 0.2137496 | 22 | 49932138 | 51018579 | 1086441 |  |

**Supplementary Table 2: Details of the ROH found in the replication dataset.** In the annotation column, “removed from analysis” indicates that the ROH spanned two chromosomes and was removed from the analysis, and “centromeric” indicates that the ROH spanned the centromere of a chromosome. Coordinates are from NCBI37.

1 3 5 7 9 11 13 15 17 19 21

1 3 5 7 9 11 13 15 17 19 21

1A)

1 3 5 7 9 11 13 15 17 19 21

1 3 5 7 9 11 13 15 17 19 21

1B)

**Supplementary Figure 1: Location within the autosome and frequency of the ROH found in A) discovery and B) replication dataset**. Dotted lines separate chromosomes.

**SUPPLEMENTARY REFERENCES**

BRODERICK, P., CHUBB, D., JOHNSON, D. C., WEINHOLD, N., FORSTI, A., LLOYD, A., OLVER, B., MA, Y., DOBBINS, S. E., WALKER, B. A., DAVIES, F. E., GREGORY, W. A., CHILDS, J. A., ROSS, F. M., JACKSON, G. H., NEBEN, K., JAUCH, A., HOFFMANN, P., MUHLEISEN, T. W., NOTHEN, M. M., MOEBUS, S., TOMLINSON, I. P., GOLDSCHMIDT, H., HEMMINKI, K., MORGAN, G. J. & HOULSTON, R. S. 2011. Common variation at 3p22.1 and 7p15.3 influences multiple myeloma risk. *Nat Genet,* 44**,** 58-61.
